# The relationship between time spent on social media and adolescent alcohol use: a longitudinal analysis of the UK Millennium Cohort Study

**DOI:** 10.1093/eurpub/ckad163

**Published:** 2023-09-12

**Authors:** Amrit Kaur Purba, Marion Henderson, Andrew Baxter, S Vittal Katikireddi, Anna Pearce

**Affiliations:** MRC/CSO Social and Public Health Sciences Unit, University of Glasgow, Glasgow, UK; MRC/CSO Social and Public Health Sciences Unit, University of Glasgow, Glasgow, UK; School of Social Work and Social Policy, University of Strathclyde, Glasgow, UK; MRC/CSO Social and Public Health Sciences Unit, University of Glasgow, Glasgow, UK; MRC/CSO Social and Public Health Sciences Unit, University of Glasgow, Glasgow, UK; MRC/CSO Social and Public Health Sciences Unit, University of Glasgow, Glasgow, UK

## Abstract

**Background:**

To estimate the effect of social media use in 14 year olds on risk of and inequalities in alcohol use and binge drinking at 17 years.

**Methods:**

Using the UK-representative Millennium Cohort Study, the relationship between time spent on social media (assessed using questionnaires [*n* = 8987] and time-use-diaries [*n* = 2520]) with frequency of alcohol use in the past month and binge drinking was estimated using adjusted odds ratios (AORs) or adjusted relative risk ratios (ARRRs). Associations within low and high parental education groups were compared to examine effect modification. Analyses accounted for pre-specified confounders, baseline outcome measures (to address reverse causality), sample design, attrition and item-missingness (through multiple imputation).

**Results:**

Questionnaire-reported time spent on social media was associated with increased risk of alcohol use and binge drinking in a dose–response manner. Compared to 1-< 30 min/day social media users, 30 min-<1 h/day users were more likely to report alcohol use ≥6 times/month (ARRR 1.62 [95% confidence interval 1.20 to 2.20]) and binge drinking (AOR 1.51 [1.22 to 1.87]), as were 1–<2 h/day users (ARRR 2.61 [1.90 to 3.58]; AOR 2.06 [1.69 to 2.52]) and ≥2 h/day users (ARRR 4.80 [3.65 to 6.32]; AOR 3.07 [2.54 to 3.70]). Social media measured by time-use-diary was associated with higher risks, although not always demonstrating a dose–response relationship. The effect of social media use (vs no-use) on binge drinking was larger in the higher (vs lower) parental education groups. Analyses repeated in complete case samples, and with adjustment for baseline outcome measures revealed consistent findings.

**Conclusions:**

Findings suggest social media use may increase risk of alcohol use and binge drinking. Regulatory action protecting adolescents from harmful alcohol-related social media content is necessary.

## Introduction

Alcohol use is a leading cause of poor health in adolescents and can lead to adverse outcomes which extend into adulthood including substance use disorders, poor mental health and reduced labour-market prospects.^1^ These health outcomes contribute to inequalities in mortality and morbidity between socioeconomic groups.^1^^,^^2^ Alcohol use is generally established during adolescence, a period of increased risk-taking and peer and social influence.^3^^,^^4^ Yet, in recent decades, a decline in adolescent drinking has been observed, suggested to result from the increased uptake and centrality of social media platforms in adolescents lives.^3^^,^^4^ However, the relationship between social media and adolescent alcohol use is complex, and despite a notable decline in adolescent alcohol use, risky drinking behaviours (e.g. binge drinking) remain high.^5^

Social media may offer adolescents opportunities to express and preserve intimacy without drinking alcohol with peers.^3^ Yet, although online activities may displace in-person interactions, the online environment may also facilitate drinking.^3^ For example, social media affords greater opportunity for in-person socializing, through its ability to maintain peer-networks, which consequently may facilitate collective drinking (in line with the Stimulation Hypothesis).^6^^,^^7^ Adolescents may use social media to present their drinking behaviour, thereby exposing their peer-network to alcohol-related content. This could promote adolescent alcohol use, in line with the Facebook Influence Model, which suggests social media may amplify existing peer influence processes.^8^ Social media has also created a new (poorly regulated) space for commercial and social marketing of alcohol practices (and other unhealthy commodities), providing new opportunities for adolescents to be exposed to pro-alcohol messages, resulting in increased consumption.^7^

This entanglement of social media and adolescent drinking cultures may contribute to the normalization of alcohol use.^9^ This is consistent with empirical evidence from Norway, the USA and the UK, which shows increased time spent on social media is associated with frequent alcohol use.^4^^,^^10^^,^^11^ However, causality and the potential for reverse causation (where those who use alcohol may be more inclined to use social media or seek out certain social media content) remains largely unaddressed.^4^^,^^10^^,^^11^ Moreover, previous research has relied on retrospective estimates of time spent via self-report questionnaires.^4^^,^^10^^,^^11^ When access to real-time objective social media data is limited, time-use-diaries might offer a useful alternative to retrospective reports, being potentially subject to less recall and response bias as they commonly employ shorter recall periods.^12^^,^^13^

The potential role of social media in widening or reducing preventable inequalities in adolescent alcohol-related diseases and deaths is largely overlooked. In line with the differential susceptibility pathway,^14^ the effects of social media on adolescent alcohol use may vary by socioeconomic circumstance (SEC). For example, it is plausible that if social media use produces greater harms to health in more socioeconomically disadvantaged adolescents, relative to those more advantaged, this could result in a widening of health inequalities.^15^^,^^16^

We aimed to estimate the effect of time spent on social media (assessed via self-report questionnaire and time-use-diary) at age 14 years on the risk of alcohol use and binge drinking at age 17 years using the UK representative Millennium Cohort Study (MCS). Addressing gaps in the evidence base, given the preventable inequalities which exist in alcohol-related diseases and deaths, we also examined if the effects of social media differ by SEC (using highest parental education as a proxy measure), with the potential to widen inequalities.

## Methods

We follow the Strengthening the Reporting of Observational Studies in Epidemiology (STROBE) guidance,^17^ and a published statistical analysis plan^18^ developed with input from a Policy Advisory Group (including patient/public representatives and stakeholders from policy, non-governmental and academic sectors); deviations are reported in Supplementary appendix SA.

### Study characteristics

**Figure 1:**
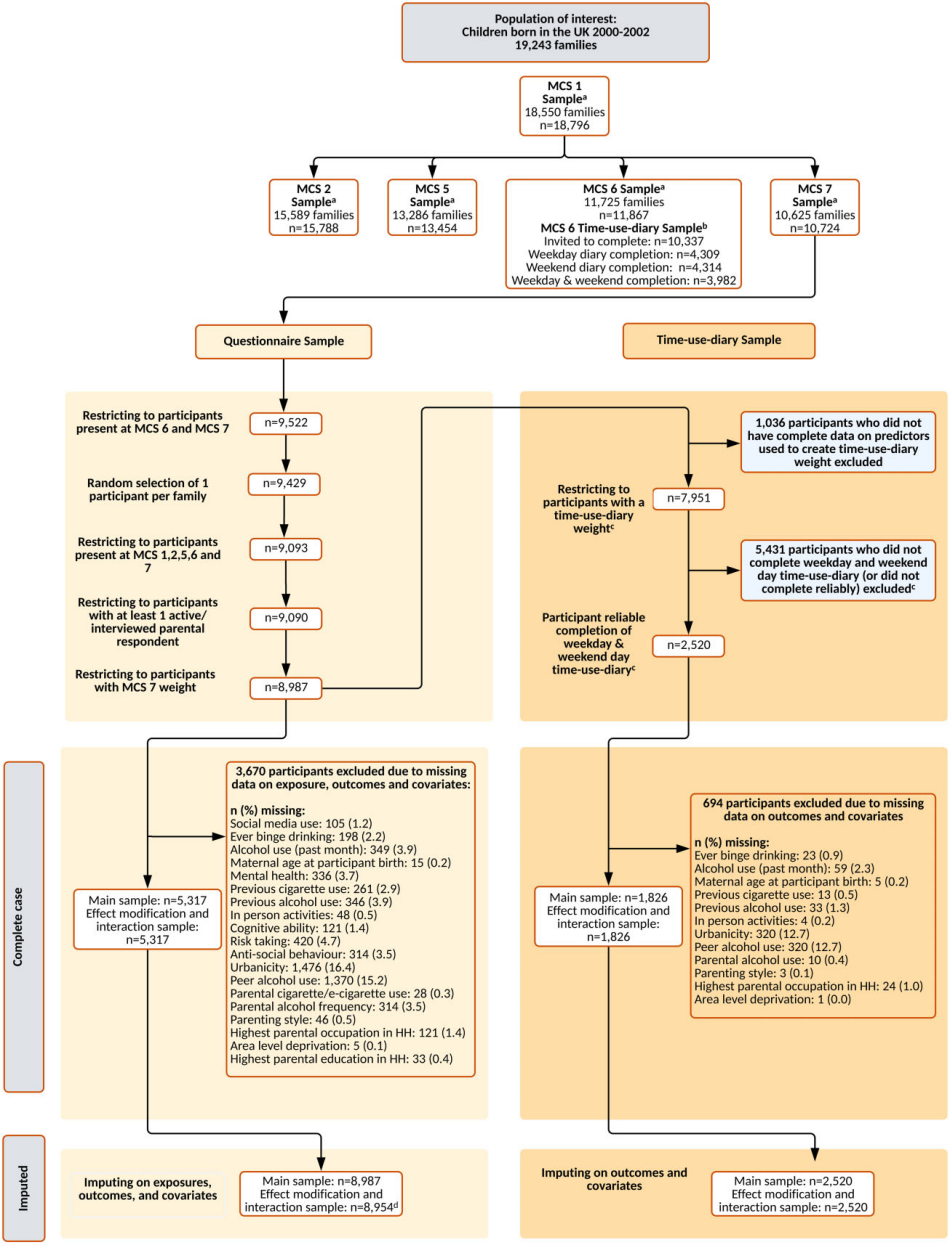
STROBE study flow diagram. ^a^MCS weights used to extrapolate back to population of interest. ^b^Time-use-diary weight created to extrapolate back to MCS 6 entire sample and combined with MCS 7 weight to extrapolate to population of interest. All predictors used to create time-use-diary weight were existing confounders to be used in analysis. ^c^Participants who had ≥5 ‘no activity recorded’ slots on a weekday, weekend or both days were deemed as having unreliable diary accounts. ^d^To facilitate inclusion of interaction between social media use and highest parental education in the imputation model for the effect modification and interaction analyses, *n* = 33 with missing data on highest parental education were excluded prior to imputation. HH, household; *n*, number of participants; MCS, Millennium Cohort Study

The MCS is a UK representative prospective cohort study of children (born September 2000–January 2002).^19^ Families were identified through Child Benefit Records, and contacted via opt-out letters from the Department for Work and Pensions. A disproportionately stratified clustered sampling design was used to over-represent children living in Wales, Scotland and Northern Ireland, disadvantaged areas, and areas with high proportions of ethnic minority groups (in the case of England).^19^ Study contact with the participant first occurred at approximately age 9 months, where data were collected from 18 796 participants. This study uses data for participants and their caregivers present in the initial survey and at ages 3 (response rate: 78.0%), 11 (69.1%), 14 (76.3%) and 17 years (74.6%).^19–21^ Triplet households were excluded. Where households contained two participants, one was randomly selected for inclusion in the analysis (figure 1). Data were downloaded from the UK Data Service (October 2021–January 2022). Ethics approval was granted for the MCS surveys; no approval was required for the current analysis.^19–21^ Information on the MCS is available from: http://www.cls.ioe.ac.uk/mcs.

## Measures

### Outcomes

Outcomes were measured at age 17 years (data collected 2018).

#### Binge drinking

Participants were asked ‘have you ever had five or more alcoholic drinks at a time?’ resulting in a dichotomous outcome variable with categories: ‘yes’ and ‘no’. A drink was defined as half a pint of lager, beer or cider, one alcopop, a small glass of wine or a measure of spirits. Each of these serving sizes is roughly equivalent to 1.5 units of alcohol (12 g pure ethanol).

#### Frequency of alcohol use in the past month

Participants were asked ‘how many times have you had an alcoholic drink in the last 4 weeks?’ with categories ranging from ‘never’ to ‘≥40 times’. Due to low frequencies in some categories, a 4-category variable was generated: ‘never’, ‘1-2 times/month’, ‘3-5 times/month’ and ‘≥6 times/month’.

### Exposures

Exposures were measured at age 14 years (data collected 2015).

#### Time spent on social media on a normal weekday during term time

Participants were asked ‘on a normal weekday during term time, how many hours do you spend on social networking or messaging sites or apps on the internet such as Facebook, Twitter and WhatsApp?’ via a self-report online questionnaire and given eight options to select from, ranging from ‘no social media use’ to ‘≥7 hours’. Due to low frequencies in the higher time categories, data were collapsed into the following: ‘no social media use’, ‘1–<30 minutes’, ‘30–<1 hour’, ‘1–<2 hours’ and ‘≥2 hours’. The reference category was ‘1–<30 minutes’, based on the threshold of potential harm in comparable studies,^4^ and because non-users are likely to be highly atypical.

#### Average time spent on social media across a normal weekday and weekend day

Participants completed the time-use-diary for two 24 hour periods (one randomly selected weekday and weekend day occurring either during term-time or during school holidays) via an online web-form, a mobile/tablet application or a paper form. For each 10 minute activity slot (144 activity slots within 24 hours), participants could select one of 44 activities, thus the diary did not account for multi-tasking. Social media use was assessed via the activity code ‘browsing and updating social networking sites (e.g. Twitter, Facebook, BBM and Snapchat)’.^22^ Adopting a comparable approach to Atkin *et al*.,^23^ diaries with ≥5 10 minutes activity slots with ‘no activity’ were deemed unreliable accounts of a complete day’s activity and were excluded from the analysis, as were participants who did not provide data on both a weekday and weekend day. Average time spent on social media across a weekday and weekend day was categorized as: ‘no social media use’, ‘1-<30 minutes’ (reference category), ‘30 minutes-<1 hour, ‘1–<2 hours’ and ‘≥2 hours’.

### Confounders

**Figure 2:**
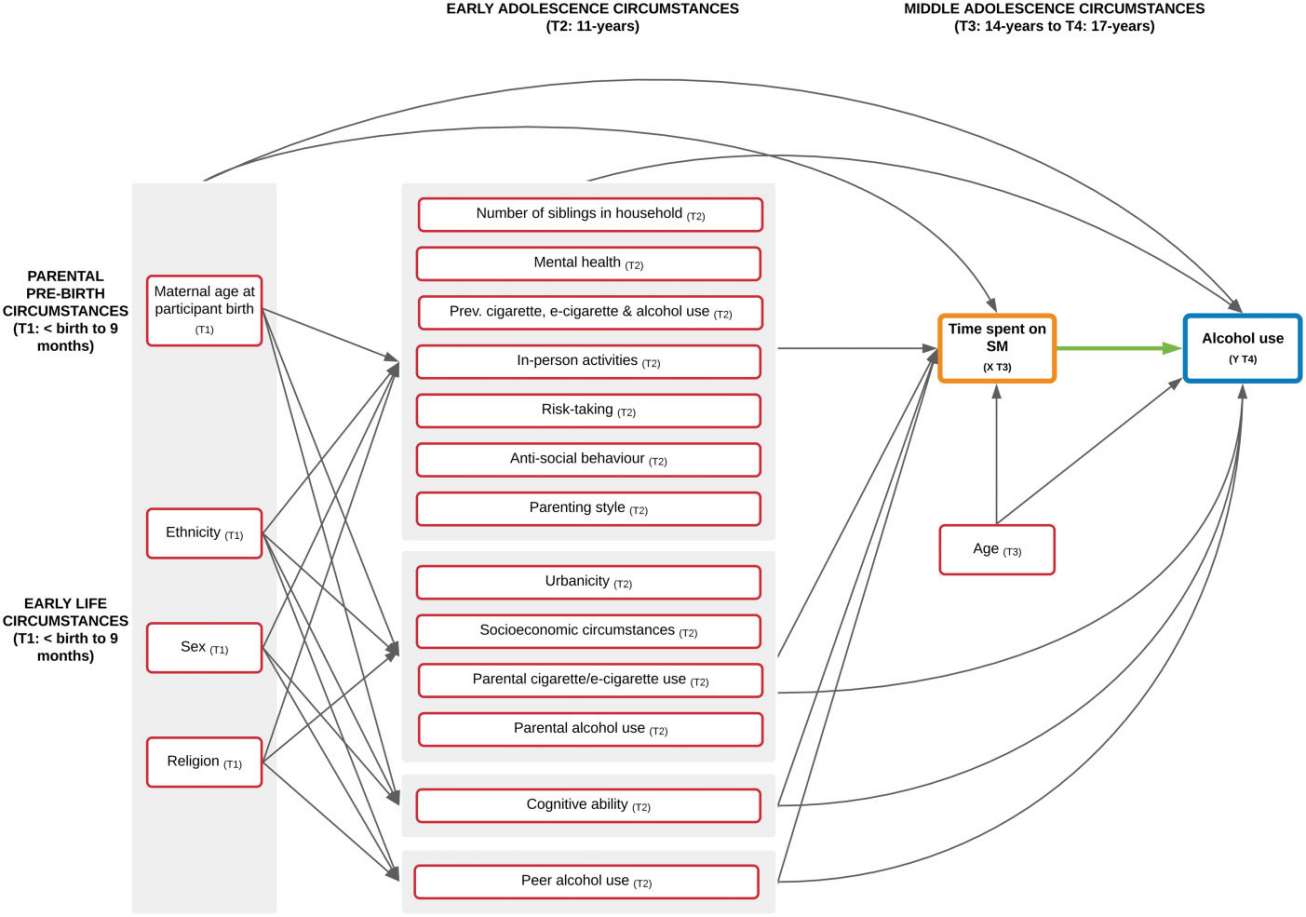
Saturated directed acyclic graph (DAG) illustrating the hypothesized relationship between social media use at 14 years and alcohol use at 17 years and the minimal sufficient adjustment set. Exposure: node denoted 'X'. Outcome: node denoted 'Y'. Observed confounders: all remaining nodes (including confounders where proxy variables are used).Arrow between Exposure (X) and Outcome (Y) indicates focal relationship under investigation. Inward and outward arrows from shaded areas pertain to all nodes within the shaded area. Confounders (information on the specific variables used to represent each confounder can be found in Supplementary appendix B): parental pre-birth and early life circumstances (T1: <birth to 9 months): maternal age at participant birth, ethnicity (6-category Census class), sex and religion. Early adolescence circumstances (T2: 11 years): number of siblings of in household, mental health (Strengths and Difficulties Questionnaire Total Difficulties), previous alcohol use, previous cigarette use (also used as a proxy measure for previous e-cigarette use), in-person activities, risk-taking (Cambridge Gambling Task), anti-social behaviour, urbanicity (Office for National Statistics Rural Urban Classification), parenting style, parental cigarette use (also used as a proxy for parental e-cigarette use), parental alcohol use, cognitive ability (British Ability Scales II Verbal Similarities), peer alcohol use and socioeconomic circumstances [household income (Organisation for Economic Co-operation and Development Income Equivalized Quintiles), family structure, parental occupation (National Statistics Socio-economic Classification), area-level deprivation (Indices of Multiple Deprivation) and parental education (National Vocational Qualification)]. Middle adolescence circumstances (T3: 14 years): age. Not shown: baseline binge drinking and frequency of alcohol use in past month (T3: 14 years) and previous social media use (T2: 11 years) adjusted for in sensitivity analyses. Socioeconomic circumstances not included in adjustment set for effect modification and interaction analysis models. SM, social media; T, timepoint

With support from our Advisory Group, subject knowledge and the existing evidence base, we prepared directed acyclic graphs (DAGs), to highlight our assumptions surrounding the causal relationship between variables of interest, and to inform our statistical approach. Confounders included parental pre-birth and early life, early and middle adolescence circumstances. The DAG shown in figure 2 presents the minimally sufficient adjustment set, identified using DAGitty software.

### Socioeconomic circumstance as an effect modifier

Parental education was used as proxy measure for SEC as it is relatively stable over time, it is strongly correlated with health behaviours and is related to other measures of SEC (e.g. income).^24^^,^^25^ Using the highest National Vocational Level of both parents in the household (where relevant), a dichotomous variable was generated representing ‘high parental education’ [International Standard Classification of Education (ISCED) 3 or A/AS/S levels or higher] and ‘low parental education’ [ISCED 2 or O level/General Certificate of Secondary Education (GSCE) grades A–C or lower].

Detail on all variables, their original format, and treatment within this study is provided in Supplementary appendix SB.

## Statistical analysis

Following a published statistical analysis plan (2022),^18^ descriptive statistics explored the association between social media and alcohol outcomes, and confounders. MCS weights accounted for the clustered sampling design and attrition. Weights were created for the time-use-diary analyses (Supplementary appendix SC). Statistical analysis was performed using *Stata.V16.*

### Imputation

Under a missing-at-random assumption, multiple imputations by chained equations were carried out in 20 datasets. Estimates were combined using Rubin’s rules.^26^ Imputation models were conducted separately for each exposure, as they have different samples and to accommodate different weights (Supplementary appendix SC). Each model included relevant outcomes, confounders, and variables used to account for sample design and attrition to age 17 years. Imputation models for effect modification and interaction analyses included an interaction between social media use and parental education so that models were compatible with the analyses. Supplementary appendix SD describes the regression models used to impute each included variable.

### Effect of social media use on frequency of alcohol use and binge drinking (primary analysis)

Odds ratios (ORs) were estimated using logistic regression to examine the association between social media use and the outcome binge drinking, before and after adjusting for confounding. Relative risk ratios were estimated using multinomial logistic regression (instead of ordinal logistic regression) for frequency of alcohol use as the proportional odds assumption was not met.^27^

#### Additional/sensitivity analyses

Analyses were repeated in complete case samples and stratified by sex. We adjusted for binge drinking and frequency of alcohol use (at 14 years), to account for possible reverse causation. These were not included in the primary analysis as they may sit on the causal pathway and consequently represent an overadjustment. We investigated questionnaire-reported social media use, replacing the reference category ‘1-<30 minutes’ with ‘no social media use’, to aid compatibility with existing evidence.^28^ We compared findings from the time-use-diary to the questionnaire exposure variable by limiting it to weekday social media use.

### Differential effect of social media use on binge drinking by socioeconomic circumstance

To examine if parental education might buffer against the potential risk of social media use on binge drinking, effect measure modification was examined by calculating risk differences (RDs; absolute differences between RDs for binge drinking by social media time category, within high and low parental education groups [baseline: low parental education)] using linear regression with robust standard errors. This method accurately estimates RDs when modelling binary outcomes.^29^ RDs assess effect measure modification on an additive scale and were our preferred approach, *a priori*, due to their greater relevance for public health.^30^

#### Additional/sensitivity analyses

As per recommendations from the STROBE guidance,^17^ and Knol and VanderWeele,^30^ interaction was examined in addition to effect measure modification (both are statistically equivalent but present results in a different way). We repeated analyses using risk ratios (RRs; estimated in Poisson regression models, with robust standard errors), which assess effect modification and interaction on the multiplicative scale,^31^ and using complete case samples. Supplementary appendix SE provides information on effect modification and interaction analyses conducted.

## Results

The questionnaire imputed sample consisted of 8987 participants (59.2% [*n* = 5317] complete data), and the time-use-diary imputed sample comprised 2520 participants (72.5% [*n* = 1826] complete data). The proportion of social media non-users in the questionnaire measure (8.4%) was considerably smaller than the time-use-diary measures (weekdays: 63.8%; averaged across weekdays and weekend days: 49.0%). These differences could be a consequence of the time-use-diaries’ inability to capture multi-tasking, resulting in a potential overestimation of social media non-users. In the questionnaire imputed sample, 13.7% of participants reported alcohol use ≥6 times/month, and 57.5% reported binge drinking. Prevalences were similar in the time-use-diary imputed sample (12.7% and 54.5%, respectively).

Supplementary appendix SF presents the characteristics of complete case and imputed samples. Imputed samples included older, and more non-White, urban, socioeconomically disadvantaged adolescents.

### Effect of social media use on frequency of alcohol use and binge drinking

**Figure 3:**
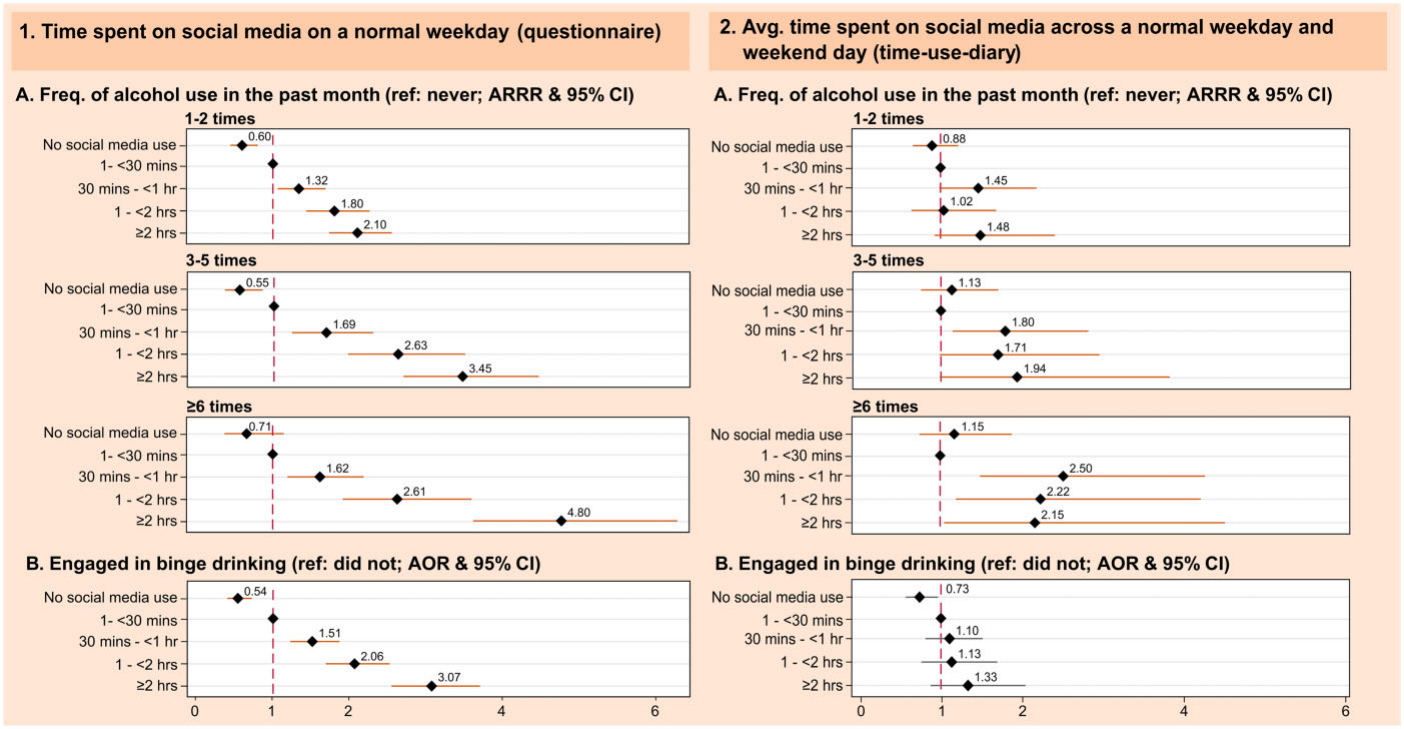
The relationship between (1) time spent on social media on a normal weekday (questionnaire) and (2) average time spent on social media across a normal weekday and weekend day (time-use-diary) with (A) frequency of alcohol use in the past month and (B) binge drinking. Questionnaire imputed sample: *n* = 8987 (weighted sample: *n* = 6175). Time-use-diary imputed sample: *n* = 2520 (weighted sample: *n* = 5005). ^a^Adjusted for sex, ethnicity, religion, peer alcohol use, parental alcohol use, parental cigarette use, parental e-cigarette use, parenting style, previous cigarette use, previous e-cigarette use, anti-social behaviour, previous alcohol use, urbanicity, age, number of siblings in household, maternal age at participant birth, in-person activities, cognitive ability, mental health, risk-taking and socioeconomic circumstances (family structure, household income, highest parental education in household, highest parental occupation in household and area-level deprivation). Values may not add up due to rounding. Avg., average; AOR, adjusted odds ratio; ARRR, adjusted relative risk ratio; CI, confidence interval; Freq., frequency; Hr/s, hour/s; Min/s, minute/s; *n*, number of participants; Ref, reference category

Questionnaire-reported time spent on social media on a normal weekday was associated with an increased risk of alcohol use and binge drinking in a dose–response manner, with higher risks seen for the more extreme outcomes (figure 3 and Supplementary appendix SG, table SG1). Among those using social media for ≥2 h/day, the risk of alcohol use 1–2 times/month (vs 1–<30 min/day users) was 2.10 (ARR, 95% confidence interval [CI] [1.73 to 2.55]). Risks were greater still for more extreme levels of drinking: 3.45 (2.68 to 4.45) and 4.80 (3.65 to 6.32) for those drinking 3–5 times/month and ≥6 times/month respectively. For binge drinking, the adjusted odds ratio (AOR) among those using social media for ≥2 h/day was 3.07 (2.54 to 3.70). Associations were potentially stronger for females (Supplementary appendix SG, table SG1). For example, females who used social media for ≥2 h/day had a greater risk of binge drinking (AOR 3.62 [2.70 to 4.87] vs 2.67 [2.11 to 3.38] for males), and alcohol use ≥6 times/month (adjusted relative risk ratio [ARRR] 6.01 [3.60 to 10.0] vs 4.34 [3.05 to 6.16] for males), though estimates were relatively imprecise.

There appeared to be a threshold effect for frequency of alcohol use in time-use-diary data, where any social media use for ≥30 min/day was associated with increased risk of alcohol use. For binge drinking, there was evidence of a weak dose–response relationship (figure 3 and Supplementary appendix SG, table SG2). No meaningful sex differences were identified, with one exception. Social media for 1–<2 h/day (vs 1–<30 min/day) presented a potential harmful effect on male alcohol use 1–2 times/month (ARRR 1.46 [0.56 to 3.80]), compared with a protective effect for females (0.89 [0.53 to 1.50]).

### Additional/sensitivity analyses

Estimates from analyses repeated in complete case samples were similar to those from imputed samples (Supplementary appendix SG, tables SG3 and SG4). In analyses comparing questionnaire and time-use-diary measures of time spent on social media on a normal weekday, there was evidence of a dose–response relationship between the time-use-diary measure of social media and binge drinking and alcohol use ≥6 times/month (Supplementary appendix SG, table SG5). This suggests the difference in effect sizes between questionnaire-reported time spent on a normal weekday and time-use-diary recorded average time spent across a normal weekday and weekend day in primary analysis may be explained by the inclusion of weekends in the time-use-diary measure. When adjusting for baseline outcome measures, dose–response relationships persisted, and estimates were similar or slightly weaker (Supplementary appendix SG, tables SG6 and SG7).

### Differential effect of social media use on binge drinking by socioeconomic circumstance

The effect of questionnaire-reported social media use on binge drinking was generally larger in the higher (compared to lower) parental education groups (table 1 and Supplementary appendix SH, table SH1). For example, the risk of binge drinking among 1–<30 min/day users (vs non-users) with high parental education was greater than those with low (adjusted risk difference [ARD]: 15.2% [8.3 to 22.1%] vs 3.4% [−7.8% to 14.7%]). In other words, the absolute difference in the ARDs between these two groups was 11.8% (−0.6% to 24.2%), indicating effect measure modification on the additive scale. Similar findings were identified for social media for 30 min–<1 h/day (effect modification measure: 15.3% [3.2 to 27.5%]), 1–<2 h/day (effect modification measure: 17.5% [5.6 to 29.3%] and ≥2 h/day (effect modification measure: 16.2% [5.2 to 27.2%]).

**Table 1:** Participant binge drinking according to time spent on social media, within strata of parental education within the questionnaire and time-use-diary imputed samples

	Questionnaire imputed sample (*n* = 8954)		Time-use-diary imputed sample (*n* = 2520)	
	High parental education	Low parental education	High parental education	Low parental education
Weighted prevalence % (observed *n* with outcome/without outcome)				
No social media use	28.1 (124/310)	31.5 (64/232)	53.3 (408/407)	47.9 (172/191)
1 - <30 min	46.4 (296/415)	36.6 (135/295)	60.8 (201/153)	51.6 (69/75)
30 min - <1 h	57.1 (430/394)	47.6 (198/303)	63.1 (157/108)	49.7 (61/64)
1 - <2 h	63.4 (575/364)	52.7 (278/341)	61.1 (107/67)	51.6 (54/48)
≥2 h	69.2 (1478/774)	61.6 (1092/857)	52.5 (58/46)	57.3 (41/34)
Unadjusted RD (95% CI; *P*-value) for time spent on social media within strata of parental education				
No social media use	Ref	Ref	Ref	Ref
1 - <30 min	18.3 (11.1 to 25.4; <0.0001)	5.1 (−8.1 to 18.3; 0.45)	7.4 (0.5 to 14.4; 0.037)	3.6 (−10.7 to 18.0; 0.62)
30 min - <1 h	28.9 (22.3 to 35.6; <0.0001)	16.1 (4.0 to 28.1; 0.009)	9.7 (2.7 to 16.8; 0.007)	1.8 (−12.5 to 16.2; 0.80)
1 - <2 h	35.3 (29.5 to 41.0; <0.0001)	21.2 (9.0 to 33.5; 0.001)	7.8 (−5.4 to 21.0; 0.25)	3.7 (−10.1 to 17.4; 0.60)
≥2 h	41.0 (35.8 to 46.3; <0.0001)	30.1 (19.2 to 41.0; <0.0001)	−0.9 (−14.1 to 12.3; 0.90)	9.4 (−8.8 to 27.6; 0.31)
Unadjusted measure of additive effect modification (95% CI; *P*-value)^a^				
No social media use	Ref		Ref	
1 - <30 min	13.2 (−1.3 to 27.7; 0.074)		3.8 (−11.9 to 19.4; 0.64)	
30 min - <1 h	12.8 (−0.8 to 26.5; 0.064)		7.9 (−7.6 to 23.4; 0.32)	
1 - <2 h	14.0 (0.7 to 27.4; 0.040)		4.1 (−16.3 to 24.6; 0.69)	
≥2 h	10.9 (−1.0 to 22.9; 0.073)		−10.3 (−32.0 to 11.4; 0.35)	
Adjusted^b^ RD (95% CI; *P*-value) for time spent on social media within strata of parental education				
No social media use	Ref	Ref	Ref	Ref
1 - <30 min	15.2 (8.3 to 22.1; <0.0001)	3.4 (−7.8 to 14.7; 0.55)	7.4 (0.8 to 14.0; 0.029)	2.4 (−9.7 to 14.4; 0.70)
30 min - <1 h	27.4 (21.2 to 33.7; <0.0001)	12.1 (1.1 to 23.1; 0.031)	10.0 (3.1 to 16.9; 0.005)	1.9 (−11.2 to 15.1; 0.77)
1 - <2 h	33.0 (26.9 to 39.2; <0.0001)	15.6 (4.7 to 26.4; 0.005)	8.4 (−4.4 to 21.2; 0.20)	4.6 (−8.8 to 18.0; 0.50)
≥2 h	40.0 (34.7 to 45.3; <0.0001)	23.8 (13.6 to 34.0; <0.0001)	6.4 (−5.9 to 18.8; 0.31)	11.7 (−2.8 to 26.2; 0.11)
Adjusted^b^ measure of additive effect modification (95% CI; *P*-value)^a^				
No social media use	Ref		Ref	
1 - <30 min	11.8 (−0.6 to 24.2; 0.063)		5.0 (−8.6 to 18.6; 0.47)	
30 min - <1 h	15.3 (3.2 to 27.5; 0.014)		8.0 (−6.4 to 22.5; 0.27)	
1 - <2 h	17.5 (5.6 to 29.3; 0.004)		3.8 (−14.8 to 22.5; 0.69)	
≥2 h	16.2 (5.2 to 27.2; 0.004)		−5.3 (−22.7 to 12.1; 0.55)	

Questionnaire imputed sample: *n* = 8954 (weighted sample: *n* = 6976). Time-use-diary imputed sample: *n* = 2520 (weighted sample: *n* = 5727).

a Measure of effect modification on an additive scale represents the size of the absolute difference between the RDs for participant binge drinking by time spent on social media, within the high parental education group compared with baseline (low parental education group).

b Adjusted for sex, ethnicity, religion, peer alcohol use, parental alcohol use, parental cigarette use, parental e-cigarette use, parenting style, previous cigarette use, previous e-cigarette use, anti-social behaviour, previous alcohol use, urbanicity, age, number of siblings in household, maternal age at participant birth, in-person activities, cognitive ability, mental health and risk-taking. Values may not add up due to rounding.

CI, confidence interval; H, hour; Min, minute; *n*, number of participants; RD, risk differences; Ref, reference category.

For the time-use-diary sample, no discernible patterns were observed (table 1 and Supplementary appendix SH, table SH1).

Investigation of interaction effects produced the same conclusions as effect modification (Supplementary appendix SH, table SH1). When conducting analyses on complete case samples, similar findings were observed (Supplementary appendix SH, table SH2). Analyses repeated using RRs showed no evidence of effect modification/interaction on the multiplicative scale (Supplementary appendix SH, tables SH3 and SH4).

## Discussion

In a UK representative, contemporary cohort, our findings suggest adolescent social media use at age 14 years increases risk of alcohol use and binge drinking in a dose–response manner. These findings generally persisted in analyses examining a range of possible biases including missing data and reverse causation. To our knowledge, no study has investigated the role of socioeconomic circumstance (SEC) as a potential effect modifier of the relationship.^32^ Using parental education as an indicator of SEC, we found the influence of social media use on binge drinking was stronger for social media users (vs non-users) with high parental education than with low when considered on the additive scale, with associations robust to adjustment for confounders.

Our findings were consistent with some of the limited longitudinal research investigating the relationship between time spent on social media and adolescent alcohol use. Among Norwegian adolescents, increased time spent was associated with increased alcohol use over time (standardized beta 0.33 [95% CI 0.26 to 0.40]; *n* = 3096).^10^ Similarly, among US adolescents an increase in social media use by 1 h in a given year was associated with a 0.06 unit increase in alcohol consumption frequency within that same year (unstandardized beta 0.06 [0.04 to 0.08]; *n* = 3612).^11^ In the single UK-based study analysing the UK Household Longitudinal Study, social media use for ≥4 h/day (in adolescents aged 16–19 years) was associated with an increase in binge drinking at follow-up, following adjustment for baseline drinking frequency (AOR 1.89 [1.01 to 3.53]; *n* = 1057).^4^ There was insufficient evidence of a relationship among those who used social media for 1–3 h/day, and when investigating use for ≥1 h/day with increased past month alcohol use. The weaker effects when compared with our study findings could be the result of the study’s use of an exposure definition which did not account for passive social media use, and instead focused on active use (defined as ‘chatting or interacting with friends through social web-sites’ within the study).^4^

Our study adds to existing evidence by investigating the relationship assessing active and passive use, adjusting for a wider range of confounders, and considering the potential impacts on health inequalities. We followed a published statistical analysis plan and investigated the impact of social media use assessed via two measurement modes.^18^ Multiple imputation accounted for item-missingness, weights accounted for attrition and additional weights were created to ensure representativeness of the time-use-diary sample. We report multiple sensitivity analyses which offer comparisons with more traditional approaches to analysis and consider bias in our methods, which our results were robust to.

The creation of directed acyclic graphs informed adjustment for a comprehensive range of confounders and the potential for reverse causality was examined, finding effects persisted when accounting for baseline measures of our outcomes. Exposure measurement occurred when participants were aged 13–15, an age range which aligns with recent research highlighting the more influential role social media plays at ages 11–15 years.^33^

Despite aiming to implement the best possible analyses for addressing the study research questions, there are factors intrinsic to the data which should be considered. Whilst we used one of the most contemporary datasets available, the MCS limited its assessment of social media to time spent and did not consider other aspects (e.g. exposure to alcohol-related content). The relatively infrequent nature of exposure and outcome timepoints limited our ability to assess the potential for reverse causation using more robust methods. Despite adjusting for a range of confounders, the potential for unmeasured or residual confounding remains. Although we included indicators for all proposed confounders as far as the data permit, there may be some we have not identified and some not adequately measured, which may lead to bias of unclear direction.

Time-use-diary completion was low (38.5%), and although weights were used to increase representativeness, the sample size was small, increasing uncertainty around presented estimates. The inability of the time-use-diary to capture multi-tasking, its potential completion during the school holidays, and it’s possible retrospective completion may have influenced reporting and underestimated social media use and overestimated non-use when compared to the questionnaire measure (findings mirrored in previous research using the time-use-diary).^23^ This misclassification, may, in part, explain the weaker associations observed for social media assessed via time-use-diary compared to the questionnaire. Yet, it is impossible to verify this in the absence of a gold standard measure. These issues could be addressed through holistically tracking social media use over multiple days across multiple devices; however, this could be a resource intensive undertaking with population-representative cohorts.^34^ Although completed individually with confidentiality emphasized, exposure and outcome measures were self-reported, thus there is potential for social desirability bias.^35^

Adolescents from less deprived backgrounds are more likely to consume alcohol,^2^ while those from more deprived backgrounds are more likely to suffer the harms of alcohol.^36^ Our investigation of parental education as an effect modifier, suggests social media use narrows inequalities in binge drinking by increasing binge drinking prevalence in more socioeconomically advantaged adolescents. This may be a result of increased exposure to alcohol-related content via social media and limited exposure to offline alcohol-related content/outlets in those more socioeconomically advantaged.^37^ Considering inequalities in alcohol harms, findings suggest increased social media use may narrow inequalities between socioeconomic groups, however not in a manner beneficial to health—instead through increasing harms in those more socioeconomically advantaged.

Future research should test these findings using more accurate social media measures, larger datasets with longer follow-up periods (and more frequent timepoints), and diverse populations. It should also seek to identify the degree to which causal relationships between the different aspects of social media use (e.g. exposure to alcohol-related content) and social media activities (e.g. passive/active) with alcohol use exist.

Social media can have several benefits to adolescent health (e.g. via online health interventions).^35^ However, the current lack of appropriate regulation of alcohol-related content may undermine positive public health messaging around alcohol-related harms. Our study strengthens calls for guidance on time spent on social media (analogous to general screentime guidance in the UK and USA),^38–40^ appropriate regulation of alcohol-related social media content and enhanced understanding of social media algorithms which drive adolescent exposure to such content, thus facilitating their active interrogation and redesign.

## Conclusion

Social media use for 30 minutes or more a day might increase frequency of alcohol use and risk of binge drinking, with evidence of dose–response relationships. Guidance on time spent on social media, regulation addressing adolescent exposure to alcohol-related social media content, and tailored social media literacy education supporting safe navigation of the social media environment should be prioritized.

## Supplementary Material

ckad163_Supplementary_Data

## Data Availability

Original MCS data are held by the UK data Service and are available on request from (https://ukdataservice.ac.uk/). Datasets accessed are listed below: Millennium Cohort Study: First Survey, 2001–3—DOI: 10.5255/UKDA-SN-4683-5 Millennium Cohort Study: Second Survey, 2003–5—DOI: 10.5255/UKDA-SN-5350-5 Millennium Cohort Study: Fifth Survey, 2012—DOI: 10.5255/UKDA-SN-7464-5 Millennium Cohort Study: Sixth Survey, 2015—DOI: 10.5255/UKDA-SN-8156-7 Millennium Cohort Study: Seventh Survey, 2018—DOI: 10.5255/UKDA-SN-8682-2 The analytic code is available in an online public repository: https://doi.org/10.5281/zenodo.7664236 https://github.com/AmritKPurba/Social_media_healthrisk_behaviours
