# Supplementary material for: The relationship between time spent on social media and adolescent alcohol use: a longitudinal analysis of the UK Millennium Cohort Study
Source: Eur J Public Health. 2023 Sep 12;33(6):1043–51. doi: 10.1093/eurpub/ckad163 (PMC10710344; doi:10.1093/eurpub/ckad163)
Supplement: ckad163_Supplementary_Data [file ckad163_supplementary_data.zip › ckad163_Supplementary_Data/ejph-2023-06-om-0290-File005.PDF]

Supplementary appendix to

The relationship between time spent on social media and adolescent alcohol use: a longitudinal analysis of the UK Millennium Cohort Study

Amrit Kaur Purba (PhD)
Marion Henderson (PhD)
Andrew Baxter (PhD)
S Vittal Katikireddi (PhD)
Anna Pearce (PhD)

Table of contents

APPENDIX-A. Deviations from published statistical analysis plan 1
APPENDIX-B. Variables used in analysis 2
APPENDIX-C. Weights used in analyses 12
Questionnaire analyses 12
Time-use-diary analyses 12
APPENDIX-D. Variables included within imputation models 14
APPENDIX-E. Differential effect of social media on binge drinking by socioeconomic circumstance: effect measure modification and interaction analyses 17
Effect modification: association between social media use and binge drinking, according to strata of parental education 17
Interaction: risk of binge drinking according to 'combinations' of social media use and parental education 17
Additional/sensitivity analyses 17
APPENDIX-F. Characteristics of imputed and complete case samples 18
APPENDIX-G. Effect of social media on frequency of alcohol use in the past month and binge drinking 24
APPENDIX-H. Differential effect of social media on binge drinking by socioeconomic circumstance 37
Assessment on the additive scale using risk differences (RDs) 37
Assessment on the multiplicative scale using risk ratios (RRs) 41
REFERENCES 45

APPENDIX-A. Deviations from published statistical analysis plan

We anticipated generating a continuous variable, representing the average time spent on social media across a weekday and weekend day (assessed via time-use-diary) adopting a fractional polynomial approach. However, following creation of the fractional polynomial, hypothesis tests comparing the generated fractional polynomial models with the linear models (where average time spent on social media across a weekday and weekend day was treated as continuous), showed no significant differences. However, a dose-response relationship was observed when this variable was categorised, suggesting some advantage of adopting a categorical variable, therefore this variable was treated as categorical in all analyses.

We intended to treat confounders number of siblings of participant in the household and age as continuous variables however due to the absence of a linear relationship with outcomes investigated, these variables were treated as categorical.

We anticipated using '1 to <30 minutes and high parental education' as the reference category for the investigation of effect measure modification and interaction. However, following assessment, the stratum with the lowest risk of binge drinking was 'no social media use and low parental education', therefore this was used as the reference category. Moreover, we planned to create a binary variable representing highest parental education in the household where National Vocational Qualification (NVQ) level 1, other academic qualifications (incl. Overseas), and none would refer to 'low parental education' and NVQ levels 2,3,4 and 5, 'high parental education'. Due to low frequencies, the following categorisation was used: low parental education (NVQ level 2, NVQ level 1, other academic qualifications (incl. Overseas), and none) and high parental education (NVQ levels 3,4 and 5).

APPENDIX-B. Variables used in analysis

Table-B1. Variables used in analysis

Variable	MCS sweep (participant age)	Ascertainment<br>Data item/survey question	Answer/category choices	Treatment in current study
Time spent on social media on a normal weekday	MCS 6 (14-years)	Self-completion time-use diary<br>Time spent browsing and updating social networking sites (e.g., Twitter, Facebook, BBM, Snapchat) on a normal weekday	Activity code<br>Browsing and updating social networking sites (e.g., Twitter, Facebook, BBM, Snapchat) on a normal weekday	5-category variable<br>On an assigned weekday, each participant recorded what they did from 04:00 to 04:00 the following day in 10-minute time slots (144 activity slots within 24 hours). The primary activity for each time slot was selected from a list of 44 predefined activity codes nested within 12 categories. The number of 10-minute activity slots recording social networking site activity on a weekday was summed to give total time (minutes) browsing and updating social networking sites on a normal weekday. The resulting continuous variable was collapsed into no social media use, 1-<30 mins, 30 mins-<60 mins, 1 hr-<2 hrs, and ≥2 hrs social media use. Adopting a similar approach to Atkin et al.[1], diaries with ≥5 10-minute activity slots with no activity indicated were excluded from the analysis as these were deemed to be unreliable accounts of a complete day's activity.
Average time spent on social media across a normal weekday and weekend day	MCS 6 (14-years)	Self-completion time-use diary<br>1. Time spent browsing and updating social networking sites (e.g., Twitter, Facebook, BBM, Snapchat) on a normal weekday<br>2. Time spent browsing and updating social networking sites (e.g., Twitter, Facebook, BBM, Snapchat) on a normal weekend day	Activity code<br>Browsing and updating social networking sites (e.g., Twitter, Facebook, BBM, Snapchat) on a normal weekday and weekend day	5-category variable<br>On an assigned weekday and weekend day, each participant recorded what they did from 04:00 to 04:00 the following day in 10-minute time slots (144 activity slots within 24 hours). The primary activity for each time slot was selected from a list of 44 predefined activity codes nested within 12 categories. The number of 10-minute activity slots recording social networking site activity on a weekday and weekend day was summed to give total time (minutes) browsing and updating social networking, this was then divided by two to give the average time spent across a weekday and weekend day. The resulting continuous variable was collapsed into no social media use, 1-<30 mins, 30 mins-<60 mins, 1 hr-<2

Variable	MCS sweep (participant age)	Ascertainment<br>Data item/survey question	Answer/category choices	Treatment in current study
				hrs, and ≥2 hrs social media use. Participants with missing data on 1or both days were classified as missing. Adopting a similar approach to Atkin et al.[1], diaries with ≥5 10-minute activity slots with no activity indicated were excluded from the analysis as these were deemed to be unreliable accounts of a complete day's activity.
Time spent on social media on a normal weekday during term time	MCS 6 (14-years)	Self-completion online questionnaire<br>Time spent on social media on a normal weekday during term time	No social media use/1-<30 mins/30-<60 mins/1 hr-<2 hrs/2 hrs-<3 hrs/3 hrs-<5 hrs/5 hrs-<7 hrs/≥7 hrs social media use/Don't know/Don't wish to answer/Not applicable	5-category variable<br>Don't know, don't wish to answer and not applicable responses were coded as missing. Categories 2 hrs-<3 hrs, 3hrs-<5 hrs, 5 hrs-<7 and ≥7 hrs were collapsed to give ≥2 hrs social media use, with no changes made to the remaining categories.<br>4-category variable<br>Don't know, don't wish to answer and not applicable responses were coded as missing. Categories 1-<30 mins and 30-<60 mins were collapsed to give 1 min-<1 hr social media use, and categories 2 hrs-<3 hrs, 3 hrs-<5 hrs, 5 hrs-<7 hrs and ≥7 hrs were collapsed to give ≥2 hrs social media use, with no changes made to the remaining categories.
Ever binge drinking	MCS 7 (17-years)	Self-completion online questionnaire<br>Has participant ever had five or more alcoholic drinks at a time? A drink is half a pint of lager, beer or cider, one alcopop, a small glass of wine, or a measure of spirits	Yes/No/Do not know/I do not wish to answer/No answer	Binary variable<br>Do not know, I do not wish to answer, and no answer responses were coded as missing. No alterations were made to the remaining categories.
Frequency of alcohol use in the past month	MCS 7 (17-years)	Self-completion online questionnaire<br>How many times participant had an alcoholic drink in the last 4 weeks?	Never/1-2 times/3-5 times/ 6-9 times/10-19 times/20-39 times/40 or more times/ Do not know/I do not wish to answer/No answer	4-category variable<br>Do not know, I do not wish to answer, and no answer responses were coded as missing. Categories were collapsed to give never, 1-2 times, 3-5 times, and 6 or more times (representing regular drinking), as per the Centre for Longitudinal Studies.[2]

Variable	MCS sweep (participant age)	Ascertainment<br>Data item/survey question	Answer/category choices	Treatment in current study
Maternal age at participant birth	MCS 1 (9 months)/ MCS 2 (3-years)	Parental interview<br>1. Respondent age at birth of participant (continuous)<br>2. Respondent ID and interview status	Continuous	Continuous variable<br>No alterations were made to variable 1. Variable 2 was used to identify the mother of the participant, and a continuous variable was generated representing maternal age at birth of the participant.
Sex	MCS 1 (9 months)/ MCS 2 (3-years)	Parental interview<br>Participant sex	Male/Female	No alterations were made to the existing variable.
Age	MCS 6 (14-years)	Parental interview<br>Participant age at last birthday	13/14/15 years	No alterations were made to the existing variable.
Ethnic group	MCS 1 (9 months)/ MCS 2 (3-years)/ MCS 3 (5-years)	Parental interview<br>Participant ethnic group (6 category Census class)	White/Mixed/Indian/ Pakistani & Bangladeshi/ Black or Black British/Other ethnic group (incl. Chinese, Other)/Refusal/Don't know/Not applicable	Binary variable<br>Refusal, don't know, and not applicable responses were coded as missing. Categories were collapsed to give White and Other (Mixed, Indian, Pakistani & Bangladeshi, Black or Black British, or Other ethnic group).
Religion	MCS 1 (9 months)/ MCS 2 (3-years)/ MCS 3 (5-years)	Parental interview<br>Main respondent religion (used as proxy for participant religion)	Christian/Muslim/Hindu/ Sikh/Jewish/Buddhist/ Other/None/Refusal/Don't know/Not applicable	Binary variable<br>Refusal, don't know and, not applicable responses were coded as missing. Categories were collapsed to give religious affiliation (Christian, Muslim, Hindu, Sikh, Jewish, Buddhist, Other) and no religious affiliation (None).

Variable	MCS sweep (participant age)	Ascertainment<br>Data item/survey question	Answer/category choices	Treatment in current study
SDQ total difficulties	MCS 5 (11-years)	Parental interview<br>Participant mental health problems (SDQ total difficulties)	Continuous score	Continuous variable<br>Calculated the total difficulties score made up of 20 items from the conduct disorders, hyperactivity/inattention, emotional and peer problem subscales of the SDQ. For each subscale variable (e.g., conduct disorders), if >2 items were completed by the parent, the scale average was imputed. Participants classified as missing are those who had some subscales completed where ≤2 items were completed or who had no SDQ information.
Previous cigarette use (also used as a proxy for previous e-cigarette use)	MCS 5 (11-years)	Self-completion online questionnaire<br>Participant ever regularly smoked tobacco products	Yes/No/Refused/Don't know/Not applicable	Binary variable<br>Refused, don't know, and not applicable responses were coded as missing. No further alterations were made to the existing variable.
Previous alcohol use	MCS 5 (11-years)	Self-completion online questionnaire<br>Participant ever had an alcoholic drink	Yes/No/No answer/Not applicable	Binary variable<br>No answer and not applicable responses were coded as missing. No further alterations were made to the existing variable.
Average days/week of in-person activity	MCS 5 (11-years)	Parental interview<br>1. Days per week participant attends a club or class to do sport or any other physical activity like swimming, gymnastics, football, or dancing<br>2. Days per week participant attends non club/class physical activities with friends/siblings (not including walking to school)	Variable 1 and 2:<br>≥5 days a week/4 days a week/3 days a week/2 days a week/1 day a week/Less often than once a week/Not at all/Don't know/ Refused/Not applicable	Continuous composite variable<br>Don't know, refused, and not applicable responses were coded as missing. Variables 1 and 2 were recoded to represent number of days of activities per week. For each variable, categories not at all and less often than once a week were combined, as engagement less than once a week would imply no engagement in a week (=0 days), 1 day a week (=1 day), 2 days a week (=2 days), 3 days a week (=3 days), 4 days a week (=4 days). As per Twenge et al.[3], and following observation of variable distributions, ≥5 days a week was coded as 6 days (the average of 5, 6, and 7 days). The sum of both frequency variables was calculated to create a composite variable representing weekly frequency of in-person activities, with no requirement to standardise as both variables were on the

Variable	MCS sweep (participant age)	Ascertainment<br>Data item/survey question	Answer/category choices	Treatment in current study
				same scale. Cronbach alpha=0.31 (as we were not measuring the same activity explicitly and instead generalising to any in-person activity, a high alpha statistic was not expected). Participants were classified as missing if they had missing data on 1/both variables used to create the composite variable.
Cognitive ability	MCS 5 (11-years)	<u>Participant assessment</u><br>BAS II Verbal Similarities - verbal reasoning and verbal knowledge	Standardised score (adjusted for age and ability)	No alterations made to the existing variable.
Risk-taking	MCS 5 (11-years)	<u>Participant assessment</u><br>CGT-risk-taking	Continuous score	No alterations made to the existing variable.
Anti-social behaviour	MCS 5 (11-years)	<u>Self-completion online questionnaire</u><br>1. Participant ever taken something from a shop without paying for it<br>2. Participant ever written things or sprayed paint on a building<br>3. Participant ever been noisy or rude in a public place<br>4. Participant ever purposely damaged anything in a public place	Variables 1-4:<br>Yes No/No answer/Not applicable	<u>Binary composite variable</u><br>No answer and not applicable responses were coded as missing. Variables 1-4 were combined to create a composite variable with categories engagement in any anti-social behaviour and engagement in no anti-social behaviours (Cronbach alpha=0.95). Participants with missing data in ≥1 variables used to create the composite variable were coded as missing.

Variable	MCS sweep (participant age)	Ascertainment<br>Data item/survey question	Answer/category choices	Treatment in current study
Number of siblings of participant in the household	MCS 5<br>(11-years)	Parental interview<br>Participant siblings in the household	Continuous	4-category variable (used in imputed time-use diary analyses)<br>Continuous variable categorised into 0, 1, 2-3 and 4-10 siblings due to issues with convergence of the imputation model.<br>5-category variable (used in remaining analyses)<br>Following observation of variable distribution, variable was categorised into 0,1,2-3,4-5 and 6-10 siblings.
Parental alcohol use	MCS 5<br>(11-years)	Parental interview<br>Parental frequency of alcohol consumption	4 or more times a week/2-3 times a week/2-4 times per month/Monthly or less/ Never/Refusal/Don't know/Not applicable	5-category variable<br>Refusal, don't know, and not applicable responses were coded as missing. No alterations were made to the remaining categories.
Parental cigarette use (also used as a proxy for parental e-cigarette use)	MCS 5<br>(11-years)	Parental interview<br>Parent current use of tobacco products	Yes/No/Not applicable	Binary composite variable<br>Not applicable responses were coded as missing. Using both main parental respondent and partner variable information (where relevant), a binary variable was generated representing smoking habits across both parents in the household with categories 1+ smoker parents and non-smoker parents. For lone parents, their smoking status was captured. For 2 parent HHs if 1 parent was missing smoking status, the present parent's smoking status was captured. If both parents were missing smoking status, the variable was classified as missing. If a lone parent was missing smoking status, the variable was classified as missing.
Parenting style	MCS 5<br>(11-years)	Parental interview<br>1. Whether parent has rules for how early or late participant may watch TV & films, use a computer, access the internet, or play electronic games<br>2. Whether parent has rules about the kinds of programmes	Yes/No/Don't know/Refused/Not applicable	Binary composite variable<br>Don't know, refused, and not applicable responses were coded as missing. Variables 1 and 2 were collapsed to generate a binary composite variable with categories parent has rules for how early or late participant can watch tv & films, use a computer, access the internet, or play electronic games or rules about the kinds of programmes or films, electronic games, internet sites they can

Variable	MCS sweep (participant age)	Ascertainment<br>Data item/survey question	Answer/category choices	Treatment in current study
		or films participant can watch, electronic games they may play, or internet sites they can access		watch, or access and parent does not have rules for either (Cronbach alpha=0.61).
Peer alcohol use	MCS 5<br>(11-years)	<u>Self-completion online questionnaire</u> How many of participants friends drink alcohol?	None of them Some of them/Most of them/All of them/Don't know/No answer/Not applicable	<u>4-category variable</u><br>Don't know, no answer and not applicable responses were coded as missing.
Urbanicity	MCS 5<br>(11-years)	<u>Parental interview</u><br>1. ONS Rural Urban Classification (2005) England & Wales<br>2. Scottish Executive Urban Rural Classification 2005/6 (2005) Scotland<br>3. ONS Rural Urban Classification (2005) Northern Ireland	Variable 1: Urban > 10k–sparse/Urban > 10k-less sparse/Town and fringe – sparse/Village, hamlet, and isolated dwellings–sparse/ Town and fringe-less sparse/Village, hamlet, and isolated dwellings-less sparse Variables 2-3: Urban/Rural	<u>Binary composite variable</u><br>Collapsed variable 1 into urban and rural and combined variables 1, 2 and 3 to generate a binary variable with categories urban and rural.
Household income	MCS 5<br>(11-years)	<u>Parental interview</u><br>Household income (OECD Income Equivalised Quintiles-UK Whole)	Bottom/Second/Third/ Fourth/Top quintile	<u>5-category variable</u><br>No alterations were made to the existing variable.
Family structure	MCS 5<br>(11-years)	<u>Parental interview</u> Family structure	Both natural parents/ Natural mother and step-parent/Natural mother and other parent or carer/ Natural mother and adoptive parent/Natural father and step-parent/ Natural father	<u>3-category variable</u><br>Categories were collapsed into natural parents, reconstituted and lone parent.

Variable	MCS sweep (participant age)	Ascertainment<br>Data item/survey question	Answer/category choices	Treatment in current study
			and other parent or carer/Adoptive parents (x2)/Foster parents (x2)/ Grandparents (x2)/ Grandmother and other parent or carer/Other parents (x2)/Natural mother only/Natural father only/Adoptive mother only/ Adoptive father only/Step-mother only/Other parent or carer only (foster/ sibling/relative)/Step-father only/Grandfather only/ Adoptive mother and step-parent	
Highest parental occupation in the household	MCS 5 (11-years)	Parental interview<br>1. NS-SEC 5 category 2. Whether respondent is in work or not	Variable 1: Managerial and professional/Intermediate/ Small employers and self-employed/Lower supervisory and technical/ Semi routine and routine/ Not applicable<br>Variable 2: Respondent is in work or on leave/ Respondent is not in work nor on leave/Not applicable	6-category composite variable<br>Variable 1 and 2 not applicable responses were coded as missing. Variable 1 and variable 2 were combined to give NS-SEC 5 category variable with an additional category representing unemployed respondents for both the main parental respondent and partner respondent (where applicable). The highest occupation of both parents in the household (where relevant) was then used for analysis. For lone parents, their occupation was captured. For 2 parent HHs if 1 parent was missing occupation, the present parent's occupation was captured. If both parents were missing occupation, the variable was classified as missing. If a lone parent was missing occupation, the variable was classified as missing.
Highest parental	MCS 5 (11-years)	Parental interview<br>NVQ equivalent of highest academic level across sweeps	NVQ level 5: higher degree/ NVQ level 4: first degree, diplomas in higher	Binary variable (used in imputed analyses)<br>The above 7-category variable was dichotomised into high parental education (NVQ level 5, level 4, and level 3) and low parental

Variable	MCS sweep (participant age)	Ascertainment<br>Data item/survey question	Answer/category choices	Treatment in current study
education in the household			education/NVQ level 3: A/AS/S levels/NVQ level 2: O level/GCSE grades A-C/ NVQ level 1: GCSE grades D-G/Other academic qualifications (incl. Overseas)/None of these/ Not applicable	education (NVQ level 2, level 1, Other academic qualifications (incl. Overseas), and None of these).<br><u>7-category variable (used in remaining analyses)</u><br>Not applicable responses were coded as missing. The highest academic education level of both parents (where relevant) was used for analysis. For lone parents, their academic qualification was captured. For 2 parent HHs, if 1 parent was missing academic qualifications, the present parent's academic qualification was captured. If both parents were missing academic qualification, the variable was classified as missing. If a lone parent was missing academic qualification, the variable was classified as missing
Area-level deprivation	MCS 5 (11-years)	<u>Parental interview</u><br>1. IMD 2004 Overall Decile England<br>2. WIMD 2005 Overall Decile Wales<br>3. SIMD 2004 Overall Decile Scotland<br>4. IMD 2004 Overall Decile Northern Ireland<br>5. Country at interview	Variables 1-4: Most deprived/10-<20%/20-<30%/ 30-<40%/40-<50%/50-<60%/ 60-<70%/70-<80%/80-<90%/ Least deprived<br>Variable 5: England/Wales/ Scotland/Northern Ireland	<u>10-category composite variable</u><br>Variables 1-4 were combined, and variable 5 was used to create indicator variables representing country.
Baseline binge drinking	MCS 6 (14-years)	<u>Self-completion online questionnaire</u><br>Has participant ever had 5 or more drinks at a time?	Yes/No/Don't want to answer/Don't know/Not applicable	<u>Binary variable</u><br>Don't want to answer, don't know and not applicable responses were coded as missing. No alterations were made to the remaining categories.

Variable	MCS sweep (participant age)	Ascertainment<br>Data item/survey question	Answer/category choices	Treatment in current study
Baseline frequency of alcohol use past year	MCS 6<br>(14-years)	Self-completion online questionnaire<br>In the last 12 months how many times has participant had an alcoholic drink?	Never/1-2 times/3-5 times/ 6-9 times/10-19 times/20-39 times/40 or more times/ Do not know/I do not wish to answer/No answer	4-category variable<br>Do not know, I do not wish to answer, and no answer responses were coded as missing. Categories were collapsed to give never, 1-2 times, 3-5 times, and 6 or more times (representing regular drinking), as per the Centre for Longitudinal Studies [2].
Previous social media use	MCS 5<br>(11-years)	Self-completion online questionnaire<br>How often participant visits a social networking website on the internet	Most days/At least once a week/At least once a month/Less often than once a month/Never/No answer/ Not applicable	4-category variable (used in imputed time-use diary effect modification and interaction analyses)<br>No answer and not applicable responses were coded as missing. Categories at least once a month and less often than once a month collapsed to give at least once a month or less than once a month. No further alterations made to remaining categories.<br>5-category variable (used in remaining analyses)<br>No answer and not applicable responses were coded as missing. No further alterations were made to existing categories.

Legend: Main parental respondent refers to the main parent of the participant completing the interview and partner refers to the partner of the main respondent. Where variable structures differ between analyses (e.g., between complete case and imputed, or between the questionnaire and time-use-diary) this was due to issues regarding imputation model convergence, thus variable structures were amended as required ensuring appropriate/meaningful categorisation. Abbreviations: BAS = British Ability Scales; BBM = Blackberry Messenger; CGT = Cambridge Gambling Task; Hrs = Hours; IMD = Indices of Multiple Deprivation; MCS = Millennium Cohort Study; NS-SEC = The National Statistics Socio-economic Classification; NVQ = National Vocational Qualification; SDQ = Strengths and Difficulties Questionnaire; OECD = Organisation for Economic Co-operation and Development, and ONS = Office for National Statistics.

APPENDIX-C. Weights used in analyses

To correct for cases having unequal probabilities of selection (due to the stratified cluster design) the Millennium Cohort Study (MCS) provides sample design weights as well as non-response weights.[4] The sample weights to be used depend on whether the analysis is restricted to data pertaining to a single country (MCS sweep 7: GOVWT1), or whether the analysis assesses all UK countries (MCS sweep 7: GOVWT2).

Questionnaire analyses

Area-level deprivation (and associated country level indicator variables) was included as a confounder within the questionnaire primary analysis models; therefore, the single country analysis weight was used. Within the effect modification and interaction analysis models, as area-level deprivation was not included as a confounder, the whole UK analysis weight was used.

Time-use-diary analyses

Due to the absence of an MCS time-use-diary weight, for the time-use-diary analyses models, we created time-use-diary analyses specific weights to make the time-use-diary sample representative of the entire sample. The weights also helped to account for participants, which were excluded due to having ≥5 'no activity' slots recorded (therefore deemed as having unreliable diary entries) and those who did not complete the time-use-diary at all.

A logistic regression approach to weighting was used when creating the weights.[5] Characteristics influencing a participant's ability to complete the time-use-diary on both the weekday and weekend day as well as complete it reliably were identified. This was achieved by entering all identified characteristics as independent variables into a logistic regression model with the binary dependent variable 1=completed the diary on both days (and reliably thus did not have ≥5 slots recorded as 'no activity') and 0=did not complete the diary on both days reliably or did not complete at all. Characteristics not identified as significant predictors of our dependent binary variable were removed in a sequential manner, until left with a model with only significant predictors (listed below):

- Sex
- Mental health (assessed via Strengths and Difficulties Questionnaire)
- Cognitive ability (assessed via British Ability Scale II Verbal Similarities)
- Risk-taking (assessed via Cambridge Gambling Task)

- Anti-social behaviour
- Parental smoking
- Household income (assessed via Organisation for Economic Co-operation and Development Income Equivalised Quintiles)
- England country indicator variable

To ensure comparability with existing MCS weights, predictors used by the MCS when creating their non-response weights (ethnicity, age, highest parental education in the household, and family structure) were also included in the model.[4,6]

In the final model, the predicted probabilities were obtained, and the inverse of the predicted probabilities calculated to obtain the time-use-diary weight. The time-use-diary weight was then multiplied by both the single country analysis weight (time-use-diary weight*GOVWT1) and whole UK analysis weight (time-use-diary weight*GOVWT2) to obtain the final weights to be used in the respective analyses (see above).

APPENDIX-D. Variables included within imputation models

Within imputation models, as recommended, predictive mean matching was used to account for the non-normal distribution of continuous variables, instead of standard regression.[7] Where convergence was not achieved for nominal categorical variables (using multinomial and ordered logit) and binary variables (using logistic), predictive mean matching was used.[7]

Table-D1 details the variables included within each of the imputation models.

Table-D1: Variables included within the imputation models

Variable	Questionnaire imputed sample (n=8,987)		Questionnaire imputed sample (n=8,954)		Time-use diary imputed sample (n=2,520)		Time-use diary imputed sample (n=2,520)	
	Primary analysis		Effect modification and interaction analysis		Primary analysis		Effect modification and interaction analysis	
	Regression model	n (%) imputed	Regression model	n (%) imputed	Regression model	n (%) imputed	Regression model	n (%) imputed
Time spent on social media on a normal weekday (time-use diary)	-	-	-	-	Complete	0 (0)	Complete	0 (0)
Average time spent on social media across a normal weekday and weekend day (time-use diary)	-	-	-	-	Complete	0 (0)	Complete	0 (0)
Time spent on social media on a normal weekday (questionnaire)	Ordered logit	105 (1.2)	Ordered logit	105 (1.2)	-	-	-	-
Ever binge drinking	Logistic	198 (2.2)	Logistic	197 (2.2)	Logistic	23 (0.9)	Logistic	23 (0.9)
Frequency of alcohol use in the past month	Ordered logit	349 (3.9)	Ordered logit	348 (3.9)	Ordered logit	59 (2.3)	Ordered logit	59 (2.3)
Maternal age at participant birth	Predictive mean matching	15 (0.2)	Predictive mean matching	13 (0.1)	Predictive mean matching	5 (0.2)	Predictive mean matching	5 (0.2)
Mental health	Predictive mean matching	336 (3.7)	Predictive mean matching	322 (0.0)	Complete	0 (0)	Complete	0 (0)

Variable	Questionnaire imputed sample (n=8,987)		Questionnaire imputed sample (n=8,954)		Time-use diary imputed sample (n=2,520)		Time-use diary imputed sample (n=2,520)	
	Primary analysis		Effect modification and interaction analysis		Primary analysis		Effect modification and interaction analysis	
	Regression model	n (%) imputed	Regression model	n (%) imputed	Regression model	n (%) imputed	Regression model	n (%) imputed
Previous cigarette use (used as proxy for previous e-cigarette use)	Logistic	261 (2.9)	Logistic	256 (2.9)	Logistic	13 (0.5)	Predictive mean matching	13 (0.5)
Previous alcohol use	Logistic	346 (3.9)	Logistic	340 (3.8)	Logistic	33 (1.3)	Predictive mean matching	33 (1.3)
Average days/week of in-person activity	Predictive mean matching	48 (0.5)	Predictive mean matching	34 (0.4)	Predictive mean matching	4 (0.2)	Predictive mean matching	4 (0.2)
Cognitive ability	Predictive mean matching	121 (1.3)	Predictive mean matching	120 (1.3)	Complete	0 (0)	Complete	0 (0)
Risk-taking	Predictive mean matching	420 (4.7)	Predictive mean matching	418 (4.7)	Complete	0 (0)	Complete	0 (0)
Anti-social behaviour	Logistic	314 (3.5)	Logistic	312 (3.5)	Complete	0 (0)	Complete	0 (0)
Peer alcohol use	Ordered logit	1,370 (15.2)	Ordered logit	1,362 (15.2)	Ordered logit	320 (1.7)	Ordered logit	320 (1.7)
Parental cigarette/e-cigarette use	Logistic	28 (0.3)	Logistic	14 (0.2)	Complete	0 (0)	Complete	0 (0)
Parental alcohol use	Predictive mean matching	314 (3.5)	Predictive mean matching	298 (3.3)	Predictive mean matching	10 (0.4)	Ordered logit	10 (0.4)
Parenting style	Logistic	46 (0.5)	Logistic	32 (0.4)	Logistic	3 (0.1)	Predictive mean matching	3 (0.1)
Urbanicity	Logistic	1,476 (16.4)	Logistic	1,470 (16.4)	Logistic	320 (12.7)	Logistic	320 (12.7)
Highest parental occupation in the household	Ordered logit	121 (1.3)	Ordered logit	107 (1.2)	Ordered logit	24 (1.0)	Ordered logit	24 (1.0)
Highest parental education in the household	Logistic	33 (0.4)	Complete: interaction by()	0 (0)[a]	Complete	0 (0)	Complete: interaction by()	0 (0)
Area-level deprivation	Ordered logit	5 (0.1)	Ordered logit	5 (0.1)	Ordered logit	1 (0.0)	Ordered logit	1 (0.0)

**Variable**	**Questionnaire imputed sample (n=8,987)**		**Questionnaire imputed sample (n=8,954)**		**Time-use diary imputed sample (n=2,520)**		**Time-use diary imputed sample (n=2,520)**	
	**Primary analysis**		**Effect modification and interaction analysis**		**Primary analysis**		**Effect modification and interaction analysis**	
	**Regression model**	**n (%) imputed**	**Regression model**	**n (%) imputed**	**Regression model**	**n (%) imputed**	**Regression model**	**n (%) imputed**
Baseline binge drinking	Logistic	237 (2.6)	-	-	Logistic	22 (0.9)	-	-
Baseline frequency of alcohol use in the past year	Ordered logit	235 (2.7)	-	-	Ordered logit	20 (0.8)	-	-
Previous social media use	Ordered logit	202 (2.2)	-	-	Ordered logit	18 (0.7)	-	-
Sex	Complete	0 (0)	Complete	0 (0)	Complete	0 (0)	Complete	0 (0)
Age	Complete	0 (0)	Complete	0 (0)	Complete	0 (0)	Complete	0 (0)
Ethnic group	Complete	0 (0)	Complete	0 (0)	Complete	0 (0)	Complete	0 (0)
Religion	Complete	0 (0)	Complete	0 (0)	Complete	0 (0)	Complete	0 (0)
Number of siblings in the household	Complete	0 (0)	Complete	0 (0)	Complete	0 (0)	Complete	0 (0)
Household income	Complete	0 (0)	Complete	0 (0)	Complete	0 (0)	Complete	0 (0)
Family structure	Complete	0 (0)	Complete	0 (0)	Complete	0 (0)	Complete	0 (0)
UK country indicator variable	Complete	0 (0)	Complete	0 (0)	Complete	0 (0)	Complete	0 (0)
GOVWT1 (MCS single country weight)	Complete	0 (0)	-	-	-	-	-	-
GOVWT2 (MCS whole UK country weight)	-	-	Complete	0 (0)	-	-	-	-
Time-use diary weight*GOVWT1 (MCS single country weight)	-	-	-	-	Complete	0 (0)	-	-
Time-use diary weight*GOVWT2 (MCS whole UK country weight)	-	-	-	-	-	-	Complete	0 (0)

Legend: [a] To facilitate inclusion of interaction between time spent on social media and highest parental education in the imputation model for effect modification and interaction analyses, n = 33 with missing data on highest parental education were excluded prior to imputation. Abbreviations: - = Not measured; MCS = Millennium Cohort Study, and n = Number of participants.

APPENDIX-E. Differential effect of social media on binge drinking by socioeconomic circumstance: effect measure modification and interaction analyses

Effect modification: association between social media use and binge drinking, according to strata of parental education

We estimated risk differences (RDs) representing the absolute difference in participant binge drinking by social media use, within the low and high parental education groups. Measures of effect modification on the additive scale represent the size of the absolute difference between RDs for binge drinking by social media use, within the high and low parental education groups, compared with the baseline (low parental education). A measure greater (or less) than zero indicates the presence of a positive (or negative) additive interaction.

Interaction: risk of binge drinking according to 'combinations' of social media use and parental education

We estimated RDs for participant binge drinking according to the combination of social media use and parental education (baseline: low parental education and no social media use (stratum with the lowest risk of binge drinking)).[8] The measure of interaction represents the size of the difference between the RD in participants with (e.g.) high parental education and 1-<30 minutes social media use, compared with the RD for participants with high parental education and no social media use, plus the RD for those with low parental education and 1-<30 minutes social media use.

Additional/sensitivity analyses

When the effect modifier is a potential cause of the outcome, as is likely for parental education and participant social media use, interaction should be examined alongside effect modification; thus, interactions were also examined.[9] A key assumption in analysis of effect modification is that the exposure (in this case, social media use) is not a cause of the effect modifier (parental education). We theorise that participant social media use does not influence parental education.[10]

We report results on both additive and multiplicative scales, in line with epidemiological recommedations.[9]

APPENDIX-F. Characteristics of imputed and complete case samples

Table-F1. Characteristics of imputed and complete case samples

	Questionnaire complete case sample (n = 5,317)		Questionnaire imputed sample (n = 8,987)		Time-use-diary complete case sample (n = 1,826)		Time-use-diary imputed sample (n = 2,520)	
**Characteristic**	**n/mean & SD**	**Weighted % (95% CI)/mean & SD[a]**	**n/mean & SD**	**Weighted % (95% CI)/mean & SD[a]**	**n/mean & SD**	**Weighted % (95% CI)/mean & SD[b]**	**n/mean & SD**	**Weighted % (95% CI)/mean & SD[b]**
**Time spent on social media on a normal weekday (questionnaire)**								
No social media use	382	7.5 (6.6 to 8.4)	734	8.4 (7.6 to 9.1)	-	-	-	-
1 - <30 mins social media use	671	12.7 (11.7 to 13.8)	1,147	12.8 (12.0 to 13.6)	-	-	-	-
30 mins - <1 hr social media use	811	15.3 (14.2 to 16.3)	1,330	14.9 (13.9 to 15.8)	-	-	-	-
1 - <2 hrs social media use	925	17.5 (16.3 to 18.7)	1,562	17.3 (16.4 to 18.3)	-	-	-	-
≥2 hrs social media use	2,528	47.1 (45.5 to 48.8)	4,214	46.6 (45.4 to 47.8)	-	-	-	-
**Time spent on social media on a normal weekday (time-use-diary)**								
No social media use	-	-	-	-	1,110	62.7 (60.2 to 65.2)	1,548	63.8 (61.7 to 65.9)
1 - <30 mins social media use	-	-	-	-	168	8.4 (7.2 to 9.8)	230	8.3 (7.2 to 9.5)
30 mins - <1 hr social media use	-	-	-	-	195	9.7 (8.3 to 11.3)	269	9.9 (8.6 to 11.1)
1 - <2 hrs social media use	-	-	-	-	197	10.1 (8.8 to 11.7)	277	10.2 (8.9 to 11.5)
≥2 hrs social media use	-	-	-	-	156	9.0 (7.4 to 11.0)	196	7.8 (6.5 to 9.1)
**Average time spent on social media across a normal weekday and weekend day (time-use-diary)**								
No social media use	-	-	-	-	831	47.2 (44.6 to 49.9)	1,178	49.0 (46.8 to 51.2)
1 - <30 mins social media use	-	-	-	-	355	18.8 (16.7 to 21.1)	497	18.8 (17.1 to 20.6)
30 mins -<1 hr social media use	-	-	-	-	294	14.5 (12.9 to 16.3)	390	14.3 (12.7 to 15.8)
1 - <2 hrs social media use	-	-	-	-	200	11.1 (9.4 to 13.1)	276	10.7 (9.2 to 12.2)
≥2 hrs social media use	-	-	-	-	146	8.4 (6.9 to 10.1)	179	7.2 (5.9 to 8.5)
**Frequency of alcohol use in the past month**								
Never	1,776	29.8 (28.2 to 31.5)	3,455	32.8 (31.2 to 34.4)	607	32.7 (30.0 to 35.5)	895	34.6 (32.1 to 37.0)
1 - 2 times	1,745	34.5 (32.9 to 36.1)	2,804	33.6 (32.1 to 35.0)	639	35.1 (32.5 to 37.9)	849	34.2 (32.1 to 36.4)

	Questionnaire complete case sample (n = 5,317)		Questionnaire imputed sample (n = 8,987)		Time-use-diary complete case sample (n = 1,826)		Time-use-diary imputed sample (n = 2,520)	
**Characteristic**	**n/mean & SD**	**Weighted % (95% CI)/mean & SD[a]**	**n/mean & SD**	**Weighted % (95% CI)/mean & SD[a]**	**n/mean & SD**	**Weighted % (95% CI)/mean & SD[b]**	**n/mean & SD**	**Weighted % (95% CI)/mean & SD[b]**
3 - 5 times	1,063	21.0 (19.7 to 22.4)	1,615	19.9 (18.7 to 21.0)	347	18.7 (16.8 to 20.9)	472	18.5 (16.8 to 20.3)
≥6 times	733	14.7 (13.5 to 16.1)	1,112	13.7 (12.8 to 14.7)	233	13.4 (11.6 to 15.5)	304	12.7 (11.0 to 14.3)
**Ever binge drinking**								
No	2,312	39.7 (37.9 to 41.5)	4,299	42.5 (40.9 to 44.2)	840	43.9 (41.2 to 46.7)	1,190	45.5 (43.1 to 48.0)
Yes	3,005	60.3 (58.5 to 62.1)	4,688	57.5 (55.8 to 59.1)	986	56.1 (53.3 to 58.8)	1,330	54.5 (52.0 to 56.9)
**Confounding variables**								
**Maternal age at participant birth**								
Mean (SD)	29.7 (5.56)	29.9 (5.50)	29.3 (5.69)	29.4 (5.67)	30.3 (5.16)	30.0 (5.30)	30.0 (5.29)	29.7 (5.46)
**Sex**								
Male	2,556	48.8 (47.2 to 50.4)	4,383	49.6 (48.4 to 50.8)	802	49.7 (46.9 to 52.5)	1,123	49.9 (47.5 to 52.3)
Female	2,761	51.2 (49.6 to 52.8)	4,604	50.4 (49.2 to 51.6)	1,024	50.3 (47.5 to 53.1)	1,397	50.1 (47.7 to 52.5)
**Age**								
13-years	1,359	25.4 (23.9 to 26.8)	2,282	25.1 (23.9 to 26.2)	467	24.7 (22.3 to 27.2)	658	26.2 (23.9 to 28.5)
14-years	3,910	73.5 (72.0 to 75.0)	6,610	73.7 (72.5 to 74.8)	1,347	74.7 (72.1 to 77.1)	1,843	73.0 (70.7 to 75.4)
15-years	48	1.1 (0.7 to 1.8)	95	1.3 (0.9 to 1.6)	12	0.6 (0.3 to 1.2)	19	0.8 (0.4 to 1.2)
**Ethnic group**								
White	4,638	93.2 (91.7 to 94.5)	7,346	91.0 (89.3 to 92.6)	1,646	92.7 (90.5 to 94.5)	2,228	92.1 (90.2 to 94.0)
Other	679	6.8 (5.5 to 8.3)	1,641	9.0 (7.4 to 10.7)	180	7.3 (5.5 to 9.5)	292	7.9 (6.0 to 9.8)
**Religion**								
Religious affiliation	3,240	59.7 (57.7 to 61.7)	5,517	58.0 (56.3 to 59.7)	1,142	59.4 (56.5 to 62.3)	1,577	58.7 (56.1 to 61.2)
No religious affiliation	2,077	40.3 (38.3 to 42.3)	3,470	42.0 (40.3 to 43.7)	684	40.6 (37.8 to 43.5)	943	41.3 (38.8 to 43.9)
**Mental health (SDQ total difficulties)**								
Mean (SD)	6.85 (5.30)	6.86 (5.43)	7.41 (5.68)	7.46 (5.89)	6.02 (4.73)	6.82 (5.36)	6.23 (4.93)	7.22 (5.81)
**Previous cigarette use (also used as proxy for previous e-cigarette use)**								

	Questionnaire complete case sample (n = 5,317)		Questionnaire imputed sample (n = 8,987)		Time-use-diary complete case sample (n = 1,826)		Time-use-diary imputed sample (n = 2,520)	
**Characteristic**	**n/mean & SD**	**Weighted % (95% CI)/mean & SD[a]**	**n/mean & SD**	**Weighted % (95% CI)/mean & SD[a]**	**n/mean & SD**	**Weighted % (95% CI)/mean & SD[b]**	**n/mean & SD**	**Weighted % (95% CI)/mean & SD[b]**
No	5,232	98.3 (97.9 to 98.7)	8,800	97.8 (97.4 to 98.3)	1,809	98.6 (97.6 to 99.2)	2,495	98.4 (97.7 to 99.2)
Yes	85	1.6 (1.3 to 2.1)	187	2.2 (1.7 to 2.6)	17	1.4 (0.8 to 2.4)	26	1.6 (0.8 to 2.3)
**Previous alcohol use**								
No	4,761	88.9 (87.8 to 89.9)	8,001	88.1 (87.2 to 89.0)	1,667	90.0 (88.2 to 91.5)	2,282	89.4 (87.8 to 91.0)
Yes	556	11.1 (10.1 to 12.2)	986	11.9 (11.0 to 12.8)	159	10.0 (8.5 to 11.8)	238	10.6 (9.0 to 12.2)
**Average days/week of in-person activity**								
Mean (SD)	3.06 (1.52)	3.14 (1.50)	2.93 (1.56)	3.04 (1.54)	3.13 (1.52)	3.15 (1.51)	3.07 (1.53)	3.07 (1.53)
**Cognitive ability**								
Mean (SD)	60.3 (9.32)	60.2 (9.36)	59.4 (10.0)	59.6 (9.87)	61.6 (8.67)	60.4 (8.77)	61.3 (8.84)	60.1 (9.02)
**Risk-taking**								
Mean (SD)	0.52 (0.17)	0.52 (0.17)	0.52 (0.17)	0.52 (0.17)	0.50 (0.17)	0.52 (0.17)	0.50 (0.17)	0.52 (0.17)
**Anti-social behaviour**								
No	4,308	80.9 (79.5 to 82.2)	7,158	79.7 (78.6 to 80.8)	1,545	80.7 (78.2 to 83.0)	2,131	80.4 (78.1 to 82.6)
Yes	1,009	19.1 (17.8 to 20.5)	1,829	20.3 (19.2 to 21.4)	281	19.3 (17.0 to 21.8)	389	19.6 (17.4 to 21.9)
**Peer alcohol use**								
None of them drink alcohol	4,892	91.7 (90.8 to 92.6)	8,244	91.3 (90.5 to 92.1)	1,709	91.7 (89.8 to 93.3)	2,342	91.2 (89.5 to 93.0)
Some of them drink alcohol	372	7.3 (6.5 to 8.2)	644	7.6 (6.9 to 8.4)	104	7.3 (5.8 to 9.3)	159	7.8 (6.1 to 9.4)
Most of them drink alcohol	29	0.6 (0.4 to 0.9)	57	0.7 (0.4 to 0.9)	10	0.8 (0.4 to 1.7)	14	0.8 (0.2 to 1.4)
All of them drink alcohol	24	0.4 (0.2 to 0.7)	40	0.4 (0.2 to 0.6)	3	0.2 (0.1 to 0.5)	4	0.2 (-0.0 to 0.3)
**Number of siblings of participant in the household**								
0	538	10.7 (9.7 to 11.7)	984	11.7 (10.9 to 12.5)	170	9.5 (8.1 to 11.1)	266	11.5 (9.9 to 13.1)
1	2,513	48.3 (46.8 to 49.8)	4,018	46.9 (45.5 to 48.3)	911	46.8 (44.2 to 49.4)	1,237	46.3 (44.0 to 48.5)
2 - 3	2,059	37.3 (35.8 to 38.8)	3,490	37.0 (35.8 to 38.3)	693	39.8 (37.0 to 42.5)	929	38.1 (35.8 to 40.5)

	Questionnaire complete case sample (n = 5,317)		Questionnaire imputed sample (n = 8,987)		Time-use-diary complete case sample (n = 1,826)		Time-use-diary imputed sample (n = 2,520)	
**Characteristic**	**n/mean & SD**	**Weighted % (95% CI)/mean & SD[a]**	**n/mean & SD**	**Weighted % (95% CI)/mean & SD[a]**	**n/mean & SD**	**Weighted % (95% CI)/mean & SD[b]**	**n/mean & SD**	**Weighted % (95% CI)/mean & SD[b]**
4 - 5	180	3.2 (2.6 to 4.0)	435	3.9 (3.3 to 4.5)	45	3.3 (2.1 to 5.0)	88	4.1 (2.9 to 5.3)
6 - 10	27	0.5 (0.3 to 0.8)	60	0.5 (0.3 to 0.6)	7	0.7 (0.3 to 1.8)		
**Parental alcohol use**								
Never	1,049	15.8 (14.5 to 17.2)	2,224	18.4 (16.9 to 19.9)	316	17.0 (15.0 to 19.2)	477	18.1 (16.1 to 20.1)
Monthly or less	1,304	24.9 (23.3 to 26.6)	2,133	24.8 (23.5 to 26.2)	420	24.0 (21.5 to 26.7)	567	23.9 (21.7 to 26.0)
2 - 4 times a month	1,165	22.8 (21.4 to 24.1)	1,854	22.5 (21.4 to 23.5)	420	22.5 (20.4 to 24.7)	586	23.1 (21.3 to 24.9)
2 - 3 times a week	1,315	26.4 (24.8 to 28.0)	2,026	24.9 (23.6 to 26.2)	473	25.1 (22.8 to 27.6)	637	24.4 (22.3 to 26.6)
≥4 times a week	484	10.1 (9.1 to 11.3)	749	9.4 (8.6 to 10.3)	197	11.4 (9.8 to 13.3)	253	10.5 (9.2 to 11.9)
**Parental cigarette use (also used as proxy for parental e-cigarette use)**								
Non-smoker parents	3,857	73.4 (71.7 to 75.1)	6,416	72.1 (70.7 to 73.5)	1,434	73.3 (70.6 to 75.9)	1,949	72.1 (69.7 to 74.5)
1+ smoker parents	1,460	26.6 (24.9 to 28.3)	2,571	27.9 (26.5 to 29.3)	392	26.7 (24.1 to 29.4)	571	27.9 (25.5 to 30.3)
**Parenting style**								
Parent has rules	5,194	97.3 (96.5 to 98.0)	8,726	97.1 (96.6 to 97.6)	1,783	97.6 (96.6 to 98.3)	2,453	97.5 (96.8 to 98.2)
Parent does not have rules	123	2.7 (2.0 to 3.5)	261	2.9 (2.4 to 3.4)	43	2.4 (1.7 to 3.5)	67	2.5 (1.8 to 3.2)
**Urbanicity**								
Urban	3,892	69.1 (65.1 to 72.8)	6,818	71.0 (67.8 to 74.2)	1,288	68.7 (64.1 to 73.1)	1,825	70.7 (66.7 to 74.7)
Rural	1,425	30.9 (27.2 to 34.9)	2,169	29.0 (25.8 to 32.2)	538	31.3 (26.9 to 35.9)	695	29.3 (25.3 to 33.3)
**Household income**								
Top quintile	1,414	29.9 (27.5 to 32.4)	2,075	27.1 (25.0 to 29.1)	594	29.7 (27.0 to 32.6)	776	28.0 (25.6 to 30.4)
Fourth quintile	1,333	26.3 (24.6 to 28.0)	2,062	25.0 (23.6 to 26.4)	522	26.7 (24.2 to 29.4)	658	25.6 (23.4 to 27.8)
Third quintile	1,168	20.9 (19.6 to 22.3)	1,883	20.6 (19.4 to 21.8)	390	21.7 (19.8 to 23.8)	533	21.6 (19.7 to 23.5)
Second quintile	831	14.3 (13.0 to 15.7)	1,514	15.4 (14.4 to 16.5)	211	13.9 (11.9 to 16.3)	312	14.5 (12.6 to 16.4)
Bottom quintile	571	8.6 (7.5 to 9.9)	1,453	11.9 (10.5 to 13.3)	109	7.9 (6.0 to 10.2)	214	10.4 (8.4 to 12.3)
**Family structure**								
Natural parents	3,879	71.2 (69.3 to 73.0	6,397	69.2 (67.6 to 70.8)	1,454	71.8 (68.7 to 74.6)	1,969	69.3 (66.8 to 71.9)

	Questionnaire complete case sample (n = 5,317)		Questionnaire imputed sample (n = 8,987)		Time-use-diary complete case sample (n = 1,826)		Time-use-diary imputed sample (n = 2,520)	
**Characteristic**	**n/mean & SD**	**Weighted % (95% CI)/mean & SD[a]**	**n/mean & SD**	**Weighted % (95% CI)/mean & SD[a]**	**n/mean & SD**	**Weighted % (95% CI)/mean & SD[b]**	**n/mean & SD**	**Weighted % (95% CI)/mean & SD[b]**
Reconstituted	470	9.4 (8.4 to 10.6)	810	10.0 (9.1 to 10.9)	126	8.3 (6.7 to 10.2)	178	9.1 (7.4 to 10.8)
Lone parent	968	19.4 (17.9 to 20.9)	1,780	20.9 (19.7 to 22.1)	246	19.9 (17.5 to 22.7)	373	21.6 (19.2 to 23.9)
**Highest parental occupation in the household**								
Managerial and professional	2,743	53.0 (50.6 to 55.3)	4,166	49.0 (46.9 to 51.1)	1,036	51.3 (47.8 to 54.8)	1,382	49.3 (46.3 to 52.4)
Intermediate	651	12.6 (11.5 to 13.7)	1,069	12.6 (11.7 to 13.5)	247	14.3 (12.4 to 16.4)	329	13.8 (12.1 to 15.5)
Small employers and self-employed	483	9.0 (8.1 to 10.0)	873	9.2 (8.4 to 10.0)	170	10.0 (8.3 to 11.9)	231	9.3 (7.8 to 10.8)
Lower supervisory and technical	205	3.6 (3.1 to 4.3)	370	3.9 (3.4 to 4.4)	48	3.3 (2.2 to 4.8)	79	3.5 (2.5 to 4.5)
Semi routine and routine	591	10.9 (9.7 to 12.3)	1,129	12.0 (10.9 to 13.0)	177	10.9 (9.0 to 13.1)	262	11.5 (9.8 to 13.3)
Unemployed	664	10.9 (9.8 to 12.1)	1,380	13.4 (12.2 to 14.5)	148	10.3 (8.4 to 12.5)	237	12.6 (10.6 to 14.5)
**Highest parental education in the household**								
NVQ level 5 - higher degree	904	17.2 (15.6 to 18.9)			350	16.0 (14.0 to 18.2)		
NVQ level 4 - first degree, diplomas in higher education	1,985	37.5 (35.7 to 39.3)	5,168	59.1 (56.9 to 61.4)	759	38.6 (35.8 to 41.5)	1,712	61.6 (58.5 to 64.7)
NVQ level 3 - A/AS/S levels	458	8.8 (7.9 to 9.8)			180	9.9 (8.5 to 11.5)		
NVQ level 2 - O level/GCSE grades A-C	1,320	25.5 (23.5 to 27.5)			392	25.4 (22.6 to 28.5)		
NVQ level 1 - GCSE grades D-G	276	4.9 (4.2 to 5.8)	3,819	40.9 (38.6 to 43.1)	73	4.7 (3.6 to 6.1)	808	38.4 (35.3 to 41.5)
Other academic qualifications (incl. Overseas)	99	1.4 (1.1 to 1.8)			26	1.6 (1.0 to 2.4)		
None of the above	275	4.7 (4.0 to 5.5)			46	3.8 (2.6 to 5.5)		
**Area-level deprivation**								
Least deprived	748	16.7 (13.9 to 19.9)	1,037	14.2 (11.8 to 16.7)	296	16.7 (13.6 to 20.4)	353	14.5 (11.7 to 17.3)
80 - <90%	648	14.5 (12.7 to 16.6)	929	13.0 (11.3 to 14.6)	243	14.9 (12.5 to 17.7)	306	13.4 (11.2 to 15.5)
70 - <80%	534	11.0 (9.5 to 12.8)	795	10.2 (8.8 to 11.6)	205	11.3 (9.2 to 13.8)	270	10.9 (8.9 to 12.8)
60 - <70%	533	10.7 (9.4 to 12.1)	809	10.3 (9.0 to 11.6)	206	11.3 (9.5 to 13.4)	275	11.4 (9.7 to 13.2)
50 - <60%	534	10.7 (9.1 to 12.6)	866	10.8 (9.3 to 12.3)	176	10.3 (8.3 to 12.8)	247	10.3 (8.4 to 12.2)

	Questionnaire complete case sample (n = 5,317)		Questionnaire imputed sample (n = 8,987)		Time-use-diary complete case sample (n = 1,826)		Time-use-diary imputed sample (n = 2,520)	
**Characteristic**	**n/mean & SD**	**Weighted % (95% CI)/mean & SD[a]**	**n/mean & SD**	**Weighted % (95% CI)/mean & SD[a]**	**n/mean & SD**	**Weighted % (95% CI)/mean & SD[b]**	**n/mean & SD**	**Weighted % (95% CI)/mean & SD[b]**
40 - <50%	525	9.2 (7.9 to 10.7)	833	9.2 (8.0 to 10.4)	190	10.3 (8.2 to 12.9)	260	10.1 (8.2 to 12.1)
30 - <40%	479	7.9 (6.8 to 9.1)	822	8.3 (7.3 to 9.3)	165	8.3 (6.7 to 10.3)	227	8.9 (7.2 to 10.6)
20 - <30%	474	7.7 (6.6 to 9.0)	901	8.6 (7.4 to 9.7)	136	7.3 (5.8 to 9.0)	213	8.5 (6.9 to 10.1)
10 - <20%	515	7.3 (6.2 to 8.5)	999	8.4 (7.3 to 9.4)	130	6.0 (4.7 to 7.5)	201	6.7 (5.4 to 7.9)
Most deprived	327	4.3 (3.5 to 5.4)	996	7.0 (5.7 to 8.4)	79	3.5 (2.6 to 4.8)	167	5.3 (3.8 to 6.8)

Legend: [a] Weighted to account attrition and sample design at the MCS 7 (17-year survey). [b] Weighted to account for time use diary non-response at MCS 6 (14-year survey) and for attrition and sample design at the MCS 7 (17-year survey). Values may not add up due to rounding. Abbreviations: - = Not measured; CI = Confidence interval; GCSE = General Certificate in Secondary Education; n = Number of participants; NVQ = National Vocational Qualifications; SD = Standard deviation, and SDQ = Strengths and Difficulties Questionnaire.

APPENDIX-G. Effect of social media on frequency of alcohol use in the past month and binge drinking

Table-G1. Time spent on social media on a normal weekday on risk of (A) frequency of alcohol use in the past month, and (B) binge drinking within the questionnaire imputed sample, stratified by sex

	**Total population (n = 8,987)**			**Males (n = 4,383)**			**Females (n = 4,604)**		
	**Weighted prevalence % (observed n with outcome)**	**RRR (95% CI)**	**ARRR (95% CI)[a]**	**Weighted prevalence % (observed n with outcome)**	**RRR (95% CI)**	**ARRR (95% CI)[a]**	**Weighted prevalence % (observed n with outcome)**	**RRR (95% CI)**	**ARRR (95% CI)[a]**
**Time spent on social media on a normal weekday (questionnaire)**									
**A. Frequency of alcohol use in the past month (ref: never)**									
**1 - 2 times**									
No social media use	23.0 (160)	0.54 (0.41 to 0.72)	0.60 (0.44 to 0.80)	22.7 (113)	0.53 (0.38 to 0.74)	0.57 (0.40 to 0.81)	23.9 (47)	0.57 (0.36 to 0.90)	0.64 (0.39 to 1.06)
1 - <30 mins	31.6 (335)	1.00	1.00	31.6 (220)	1.00	1.00	31.7 (115)	1.00	1.00
30 mins - <1 hr	33.7 (404)	1.32 (1.06 to 1.64)	1.32 (1.05 to 1.67)	33.6 (243)	1.37 (1.01 to 1.84)	1.34 (0.98 to 1.82)	33.9 (160)	1.28 (0.92 to 1.78)	1.37 (0.95 to 1.97)
1 - <2 hrs	35.9 (515)	1.79 (1.44 to 2.22)	1.80 (1.43 to 2.26)	33.8 (250)	1.68 (1.27 to 2.22)	1.69 (1.24 to 2.29)	38.2 (265)	1.98 (1.40 to 2.82)	2.03 (1.40 to 2.95)
≥2 hrs	35.0 (1,391)	1.96 (1.65 to 2.32)	2.10 (1.73 to 2.55)	30.9 (431)	1.75 (1.34 to 2.28)	1.87 (1.39 to 2.50)	37.4 (959)	2.21 (1.66 to 2.94)	2.41 (1.77 to 3.30)
**3 - 5 times**									
No social media use	10.0 (35)	0.48 (0.32 to 0.73)	0.55 (0.36 to 0.85)	9.7 (44)	0.48 (0.28 to 0.82)	0.57 (0.33 to 0.99)	8.9 (17)	0.48 (0.25 to 0.91)	0.54 (0.27 to 1.09)
1 - <30 mins	15.7 (89)	1.00	1.00	14.8 (93)	1.00	1.00	14.0 (46)	1.00	1.00
30 mins - <1 hr	19.2 (151)	1.59 (1.18 to 2.13)	1.69 (1.24 to 2.30)	19.8 (146)	1.72 (1.14 to 2.60)	1.84 (1.19 to 2.85)	16.9 (75)	1.44 (0.92 to 2.24)	1.57 (0.97 to 2.54)
1 - <2 hrs	22.8 (204)	2.42 (1.86 to 3.15)	2.63 (1.98 to 3.50)	22.7 (172)	2.41 (1.73 to 3.35)	2.58 (1.77 to 3.76)	22.0 (140)	2.57 (1.65 to 4.02)	2.70 (1.67 to 4.37)
≥2 hrs	24.1 (584)	2.75 (2.18 to 3.48)	3.45 (2.68 to 4.45)	22.9 (324)	2.76 (2.00 to 3.81)	3.50 (2.48 to 4.93)	22.5 (559)	3.00 (2.03 to 4.43)	3.42 (2.23 to 5.24)
**≥6 times**									
No social media use	8.5 (28)	0.63 (0.38 to 1.04)	0.71 (0.42 to 1.20)	9.8 (38)	0.65 (0.37 to 1.13)	0.77 (0.43 to 1.38)	2.7 (7)	0.38 (0.14 to 1.01)	0.42 (0.16 to 1.11)
1 - <30 mins	10.8 (61)	1.00	1.00	11.1 (69)	1.00	1.00	5.4 (19)	1.00	1.00

30 mins - <1 hr	12.9 (98)	1.53 (1.14 to 2.05)	1.62 (1.20 to 2.20)	13.4 (98)	1.55 (1.10 to 2.19)	1.57 (1.09 to 2.26)	8.1 (39)	1.80 (1.02 to 3.17)	2.00 (1.11 to 3.61)
1 - <2 hrs	14.9 (132)	2.28 (1.69 to 3.08)	2.61 (1.90 to 3.58)	16.3 (125)	2.30 (1.63 to 3.25)	2.34 (1.59 to 3.42)	10.1 (68)	3.09 (1.75 to 5.45)	3.41 (1.90 to 6.14)
≥2 hrs	17.3 (414)	3.26 (2.51 to 4.24)	4.80 (3.65 to 6.32)	22.3 (303)	3.58 (2.58 to 4.96)	4.34 (3.05 to 6.16)	13.9 (345)	4.84 (2.94 to 7.97)	6.01 (3.60 to 10.0)
	**Weighted prevalence % (observed n with outcome)**	**OR (95% CI)**	**AOR (95% CI)[a]**	**Weighted prevalence % (observed n with outcome)**	**OR (95% CI)**	**AOR (95% CI)[a]**	**Weighted prevalence % (observed n with outcome)**	**OR (95% CI)**	**AOR (95% CI)[a]**
**B. Binge drinking (ref: no binge drinking**									
No social media use	28.7 (188)	0.51 (0.39 to 0.67)	0.54 (0.41 to 0.72)	32.4 (146)	0.50 (0.36 to 0.69)	0.56 (0.39 to 0.80)	19.5 (42)	0.46 (0.29 to .075)	0.51 (0.31 to 0.84)
1 - <30 mins	43.9 (433)	1.00	1.00	48.8 (319)	1.00	1.00	34.4 (114)	1.00	1.00
30 mins - <1 hr	53.1 (630)	1.44 (1.18 to 1.77)	1.51 (1.22 to 1.87)	59.3 (429)	1.53 (1.19 to 1.97)	1.53 (1.18 to 2.00)	43.2 (200)	1.45 (1.04 to 2.02)	1.58 (1.11 to 2.50)
1 - <2 hrs	60.5 (857)	1.96 (1.62 to 2.36)	2.06 (1.69 to 2.52)	67.3 (503)	2.16 (1.70 to 2.74)	2.16 (1.66 to 2.80)	53.2 (353)	2.17 (1.58 to 2.98)	2.18 (1.56 to 3.03)
≥2 hrs	66.7 (2,581)	2.55 (2.15 to 3.03)	3.07 (2.54 to 3.70)	70.4 (971)	2.50 (1.99 to 3.13)	2.67 (2.11 to 3.38)	64.5 (1,609)	3.47 (2.64 to 4.56)	3.62 (2.70 to 4.87)

Legend: Questionnaire imputed sample: n = 8,987 (weighted sample: n = 6,175). [a] Adjusted for sex, ethnicity, religion, peer alcohol use, parental alcohol use, parental cigarette use, parental e-cigarette use, parenting style, previous cigarette use, previous e-cigarette use, anti-social behaviour, previous alcohol use, urbanicity, age, number of siblings in household, maternal age at participant birth, in-person activities, cognitive ability, mental health, risk-taking, and socioeconomic circumstances (family structure, household income, highest parental education in household, highest parental occupation in household, and area-level deprivation). Total population estimates adjusted for sex; sex stratified estimates are not. Values may not add up due to rounding. Abbreviations: AOR = Adjusted odds ratio; ARRR = Adjusted relative risk ratio; CI = Confidence interval; Hr/s = Hour/s; Min/s = Minute/s; n = Number of participants; OR = Odds ratio; Ref = Reference category, and RRR = Relative risk ratio.

Table-G2. Average time spent on social media across a normal weekday and weekend day on risk of (A) frequency of alcohol use in the past month, and (B) binge drinking within the time-use-diary imputed sample, stratified by sex

	Total population (n = 2,520)			Males (n = 1,123)			Females (n = 1,397)		
	**Weighted prevalence % (observed n with outcome)**	**RRR (95% CI)**	**ARRR (95% CI)[a]**	**Weighted prevalence % (observed n with outcome)**	**RRR (95% CI)**	**ARRR (95% CI)[a]**	**Weighted prevalence % (observed n with outcome)**	**RRR (95% CI)**	**ARRR (95% CI)[a]**
**Average time spent on social media across a normal weekday and weekend day (time-use-diary)**									
**A. Frequency of alcohol use in the past month (ref: never)**									
**1-2 times**									
No social media use	31.7 (367)	0.80 (0.59 to 1.09)	0.88 (0.64 to 1.20)	30.8 (216)	1.06 (0.67 to 1.68)	1.11 (0.68 to 1.79)	33.5 (151)	0.66 (0.45 to 0.99)	0.67 (0.44 to 1.01)
1 - <30 mins	37.2 (183)	1.00	1.00	31.6 (64)	1.00	1.00	41.6 (119)	1.00	1.00
30 mins - <1 hr	38.2 (149)	1.42 (0.93 to 2.17)	1.45 (0.97 to 2.17)	34.9 (42)	1.60 (0.79 to 3.25)	1.56 (0.79 to 3.11)	39.9 (107)	1.26 (0.79 to 2.01)	1.37 (0.86 to 2.20)
1 - <2 hrs	31.9 (85)	1.03 (0.64 to 1.64)	1.02 (0.62 to 1.67)	27.9 (22)	1.15 (0.45 to 2.93)	1.46 (0.56 to 3.80)	34.0 (63)	0.91 (0.56 to 1.46)	0.89 (0.53 to 1.50)
≥2 hrs	39.0 (65)	1.26 (0.78 to 2.03)	1.48 (0.91 to 2.40)	30.8 (11)	1.23 (0.44 to 3.46)	2.03 (0.71 to 5.79)	41.2 (54)	1.10 (0.64 to 1.91)	1.19 (0.66 to 2.13)
**3-5 times**									
No social media use	17.7 (210)	1.00 (0.69 to 1.45)	1.13 (0.75 to 1.71)	17.4 (126)	1.39 (0.78 to 2.48)	1.65 (0.86 to 3.14)	18.2 (85)	0.79 (0.49 to 1.26)	0.75 (0.46 to 1.24)
1 - <30 mins	16.7 (88)	1.00	1.00	13.7 (28)	1.00	1.00	19.0 (59)	1.00	1.00
30 mins - <1 hr	20.3 (79)	1.69 (1.09 to 2.60)	1.80 (1.14 to 2.82)	18.5 (22)	1.96 (0.87 to 4.38)	1.77 (0.76 to 4.15)	21.2 (57)	1.47 (0.86 to 2.50)	1.53 (0.87 to 2.71)
1 - <2 hrs	22.2 (63)	1.60 (0.95 to 2.68)	1.71 (0.98 to 2.96)	22.9 (22)	2.19 (0.82 to 5.85)	3.44 (1.27 to 9.33)	21.8 (40)	1.27 (0.72 to 2.24)	1.24 (0.67 to 2.32)
≥2 hrs	20.0 (33)	1.44 (0.76 to 2.75)	1.94 (0.98 to 3.83)	24.0 (4)	2.22 (0.40 to 12.34)	2.66 (0.71 to 9.98)	18.9 (29)	1.11 (0.61 to 2.03)	1.57 (0.76 to 3.24)
**≥6 times**									
No social media use	12.4 (140)	1.18 (0.75 to 1.84)	1.15 (0.71 to 1.86)	14.6 (99)	1.11 (0.60 to 2.06)	1.16 (0.61 to 2.22)	8.5 (41)	1.03 (0.58 to 1.85)	1.12 (0.58 to 2.17)
1 - <30 mins	9.9 (50)	1.00	1.00	14.3 (26)	1.00	1.00	6.8 (24)	1.00	1.00

30 mins - <1 hr	15.5 (53)	2.15 (1.25 to 3.68)	2.50 (1.47 to 4.25)	18.6 (23)	1.88 (0.84 to 4.20)	1.87 (0.84 to 4.13)	13.8 (30)	2.69 (1.29 to 5.62)	3.17 (1.58 to 6.34)
1 - <2 hrs	15.9 (39)	1.91 (1.04 to 3.48)	2.22 (1.17 to 4.20)	18.2 (13)	1.67 (0.57 to 4.85)	2.03 (0.66 to 6.22)	14.6 (26)	2.40 (1.19 to 4.82)	2.73 (1.34 to 5.57)
≥2 hrs	11.1 (22)	1.33 (0.67 to 2.65)	2.15 (1.03 to 4.51)	13.2 (5)	1.16 (0.32 to 4.20)	1.91 (0.57 to 6.46)	10.5 (17)	1.73 (0.77 to 3.89)	2.59 (1.04 to 6.46)
	**Weighted prevalence % (observed n with outcome)**	**OR (95% CI)**	**AOR (95% CI)[a]**	**Weighted prevalence % (observed n with outcome)**	**OR (95% CI)**	**AOR (95% CI)[a]**	**Weighted prevalence % (observed n with outcome)**	**OR (95% CI)**	**AOR (95% CI)[a]**
**B. Binge drinking (ref: no binge drinking)**									
No social media use	50.8 (581)	0.77 (0.60 to 1.00)	0.73 (0.55 to 0.96)	55.6 (385)	0.79 (0.53 to 1.16)	0.91 (0.59 to 1.40)	42.0 (197)	0.62 (0.45 to 0.86)	0.62 (0.44 to 0.88)
1 - <30 mins	57.2 (270)	1.00	1.00	61.5 (111)	1.00	1.00	53.9 (158)	1.00	1.00
30 mins - <1 hr	58.3 (218)	1.05 (0.78 to 1.42)	1.10 (0.80 to 1.52)	64.4 (78)	1.13 (0.66 to 1.95)	1.11 (0.62 to 1.98)	55.2 (140)	1.05 (0.71 to 1.56)	1.06 (0.70 to 1.60)
1 - <2 hrs	59.6 (162)	1.10 (0.76 to 1.61)	1.13 (0.75 to 1.70)	61.7 (51)	1.01 (0.49 to 2.06)	1.21 (0.59 to 2.48)	58.4 (111)	1.20 (0.80 to 1.81)	1.17 (0.73 to 1.86)
≥2 hrs	57.1 (99)	1.00 (0.66 to 1.51)	1.33 (0.87 to 2.05)	58.8 (17)	0.90 (0.33 to 2.46)	1.24 (0.51 to 3.03)	56.7 (82)	1.12 (0.72 to 1.74)	1.27 (0.78 to 2.06)

Legend: Time-use-diary imputed sample: n = 2,520 (weighted sample: n = 5,005). [a] Adjusted for sex, ethnicity, religion, peer alcohol use, parental alcohol use, parental cigarette use, parental e-cigarette use, parenting style, previous cigarette use, previous e-cigarette use, anti-social behaviour, previous alcohol use, urbanicity, age, number of siblings in household, maternal age at participant birth, in-person activities, cognitive ability, mental health, risk-taking, and socioeconomic circumstances (family structure, household income, highest parental education in household, highest parental occupation in household, and area-level deprivation). Total population estimates adjusted for sex; sex stratified estimates are not. Values may not add up due to rounding. Abbreviations: AOR = Adjusted odds ratio; ARRR = Adjusted relative risk ratio; CI = Confidence interval; Hr/s = Hour/s; Min/s = Minute/s; n = Number of participants; OR = Odds ratio; Ref = Reference category, and RRR = Relative risk ratio.

Table-G3. Time spent on social media on a normal weekday on risk of (A) frequency of alcohol use in the past month, and (B) binge drinking within the questionnaire complete case sample

	Questionnaire complete case sample (n = 5,317)		
	**Weighted prevalence % (observed n with outcome)**	**RRR (95% CI)**	**ARRR (95% CI)[a]**
**Time spent on social media on a normal weekday (questionnaire)**			
**A. Frequency of alcohol use in the past month (ref: never)**			
**1-2 times**			
No social media use	25.6 (98)	0.60 (0.44 to 0.83)	0.62 (0.45 to 0.86)
1 - <30 mins	31.6 (212)	1.00	1.00
30 mins - <1 hr	34.5 (260)	1.36 (1.06 to 1.75)	1.33 (1.02 to 1.72)
1 - <2 hrs	35.9 (312)	1.80 (1.36 to 2.38)	1.79 (1.37 to 2.33)
≥2 hrs	36.1 (863)	2.12 (1.70 to 2.63)	2.22 (1.76 to 2.81)
**3-5 times**			
No social media use	10.0 (35)	0.48 (0.30 to 0.74)	0.51 (0.33 to 0.80)
1 - <30 mins	15.7 (89)	1.00	1.00
30 mins - <1 hr	19.2 (151)	1.53 (1.08 to 2.17)	1.56 (1.08 to 2.56)
1 - <2 hrs	22.8 (204)	2.31 (1.67 to 3.19)	2.54 (1.81 to 3.56)
≥2 hrs	24.1 (584)	2.85 (2.14 to 3.78)	3.61 (2.72 to 4.80)
**≥6 times**			
No social media use	8.5 (28)	0.58 (0.32 to 1.07)	0.60 (0.34 to 1.06)
1 - <30 mins	10.8 (61)	1.00	1.00
30 mins - <1 hr	12.9 (98)	1.49 (1.07 to 2.07)	1.49 (1.07 to 2.09)
1 - <2 hrs	14.9 (132)	2.19 (1.49 to 3.20)	2.55 (1.72 to 3.79)
≥2 hrs	17.3 (414)	2.97 (2.23 to 3.97)	4.45 (3.29 to 6.02)
	**Weighted prevalence % (observed n with outcome)**	**OR (95% CI)**	**AOR (95% CI)[a]**
**B. Binge drinking (ref: no binge drinking)**			
No social media use	29.3 (109)	0.47 (0.34 to 0.64)	0.46 (0.33 to 0.64)
1 - <30 mins	46.9 (284)	1.00	1.00
30 mins - <1 hr	56.5 (430)	1.47 (1.17 to 1.83)	1.52 (1.21 to 1.92)
1 - <2 hrs	62.8 (549)	1.91 (1.52 to 2.39)	2.07 (1.65 to 2.60)
≥2 hrs	69.1 (1,633)	2.53 (2.10 to 3.05)	3.21 (2.64 to 3.91)

Legend: Questionnaire complete case sample: n = 5,317 (weighted sample: n = 3,818). [a] Adjusted for sex, ethnicity, religion, peer alcohol use, parental cigarette use, parental e-cigarette use, parenting style, previous cigarette use, previous e-cigarette use, anti-social behaviour, previous alcohol use, urbanicity, age, number of siblings in household, maternal age at participant birth, in-person activities, cognitive ability, mental health, risk-taking, and socioeconomic circumstances (family structure, household income, highest parental education in household, highest parental occupation in household, and area-level deprivation). Values may not add up due to rounding. Abbreviations: AOR = Adjusted odds ratio; ARRR = Adjusted relative risk ratio; CI = Confidence interval; Hr/s = Hour/s; Min/s = Minute/s; n = Number of participants; OR = Odds ratio; Ref = Reference category, and RRR = Relative risk ratio.

Table-G4. Average time spent on social media across a normal weekday and weekend day on risk of (A) frequency of alcohol use in the past month, and (B) binge drinking within the time-use-diary complete case sample

	Time-use-diary complete case sample (n = 1,826)		
	**Weighted prevalence % (observed n with outcome)**	**RRR (95% CI)**	**ARRR (95% CI)[a]**
**Average time spent on social media across a normal weekday and weekend day (time-use-diary)**			
**A. Frequency of alcohol use in the past month (ref: never)**			
**1-2 times**			
No social media use	32.5 (269)	0.85 (0.58 to 1.24)	0.96 (0.66 to 1.40)
1 - <30 mins	37.3 (136)	1.00	1.00
30 mins - <1 hr	40.9 (116)	1.51 (0.95 to 2.41)	1.61 (1.03 to 2.51)
1 - <2 hrs	31.6 (64)	1.11 (0.62 to 1.97)	1.06 (0.61 to 1.84)
≥2 hrs	40.0 (54)	1.21 (0.70 to 2.08)	1.45 (0.84 to 2.51)
**3-5 times**			
No social media use	17.4 (142)	0.97 (0.61 to 1.52)	1.04 (0.65 to 1.66)
1 - <30 mins	17.5 (67)	1.00	1.00
30 mins - <1 hr	20.2 (62)	1.60 (0.97 to 2.64)	1.66 (1.00 to 2.77)
1 - <2 hrs	25.0 (51)	1.87 (0.99 to 3.56)	1.72 (0.92 to 3.19)
≥2 hrs	18.2 (25)	1.17 (0.55 to 2.49)	1.36 (0.69 to 2.71)
**≥6 times**			
No social media use	14.2 (110)	1.32 (0.76 to 2.29)	1.34 (0.76 to 2.38)
1 - <30 mins	10.5 (38)	1.00	1.00
30 mins - <1 hr	13.7 (39)	1.81 (1.00 to 3.28)	2.27 (1.26 to 4.09)
1 - <2 hrs	16.8 (29)	2.11 (1.01 to 4.40)	2.29 (1.08 to 4.86)
≥2 hrs	11.0 (17)	1.18 (0.54 to 2.59)	1.99 (0.90 to 4.43)
	**Weighted prevalence % (observed n with outcome)**	**OR**	**AOR[a]**
**B. Binge drinking (ref: no binge drinking**			
No social media use	52.7 (415)	0.74 (0.56 to 1.00)	0.72 (0.53 to 0.99)
1 - <30 mins	60.0 (203)	1.00	1.00
30 mins - <1 hr	52.3 (166)	0.89 (0.63 to 1.27)	0.99 (0.68 to 1.44)
1 - <2 hrs	62.3 (123)	1.10 (0.69 to 1.77)	1.16 (0.73 to 1.85)
≥2 hrs	55.7 (79)	0.84 (0.54 to 1.31)	1.13 (0.72 to 1.78)

Legend: Time-use-diary complete case sample: n = 1,826 (weighted sample: n = 3,601). [a] Adjusted for sex, ethnicity, religion, peer alcohol use, parental alcohol use, parental cigarette use, parental e-cigarette use, parenting style, previous cigarette use, previous e-cigarette use, anti-social behaviour, previous alcohol use, urbanicity, age, number of siblings in household, maternal age at participant birth, in-person activities, cognitive ability, mental health, risk-taking, and socioeconomic circumstances (family structure, household income, highest parental education in household, highest parental occupation in household, and area-level deprivation). Values may not add up due to rounding. Abbreviations: AOR = Adjusted odds ratio; ARRR = Adjusted relative risk ratio; CI = Confidence interval; Hr/s = Hour/s; Min/s = Minute/s; n = Number of participants; OR = Odds ratio; Ref = Reference category, and RRR = Relative risk ratio.

Table-G5. Comparison of estimates for time spent on social media on a normal weekday on risk of (A) frequency of alcohol use in the past month, and (B) binge drinking within the questionnaire and time-use-diary imputed samples

	**Questionnaire imputed sample (n = 8,987)**			**Time-use-diary imputed sample (n = 2,520)**		
	**Weighted prevalence % (observed n with outcome)**	**RRR (95% CI)**	**ARRR (95% CI)[a]**	**Weighted prevalence % (observed n with outcome)**	**RRR (95% CI)**	**ARRR (95% CI)[a]**
**Time spent on social media on a normal weekday**						
**A. Frequency of alcohol use in the past month (ref: never)**						
**1-2 times**						
No social media use	23.0 (160)	0.54 (0.41 to 0.72)	0.60 (0.44 to 0.80)	32.6 (505)	0.99 (0.68 to 1.44)	1.09 (0.74 to 1.61)
1 - <30 mins	31.6 (335)	1.00	1.00	33.2 (78)	1.00	1.00
30 mins - <1 hr	33.7 (404)	1.32 (1.06 to 1.64)	1.32 (1.05 to 1.67)	40.5 (98)	1.53 (0.91 to 2.56)	1.46 (0.86 to 2.50)
1 - <2 hrs	35.9 (515)	1.79 (1.44 to 2.22)	1.80 (1.43 to 2.26)	36.4 (93)	1.54 (0.90 to 2.61)	1.76 (1.00 to 3.10)
≥2 hrs	35.0 (1,391)	1.96 (1.65 to 2.32)	2.10 (1.73 to 2.55)	38.2 (75)	1.53 (0.96 to 2.45)	1.73 (1.03 to 2.90)
**3-5 times**						
No social media use	9.4 (61)	0.48 (0.32 to 0.73)	0.55 (0.36 to 0.85)	17.6 (282)	1.06 (0.65 to 1.74)	1.26 (0.74 to 2.15)
1 - <30 mins	14.5 (139)	1.00	1.00	16.7 (40)	1.00	1.00
30 mins - <1 hr	18.7 (221)	1.59 (1.18 to 2.13)	1.69 (1.24 to 2.30)	20.0 (54)	1.50 (0.85 to 2.64)	1.48 (0.79 to 2.79)
1 - <2 hrs	22.4 (311)	2.42 (1.86 to 3.15)	2.63 (1.98 to 3.50)	23.4 (64)	1.96 (1.08 to 3.55)	2.42 (1.25 to 4.66)
≥2 hrs	22.7 (883)	2.75 (2.18 to 3.48)	3.45 (2.68 to 4.45)	19.4 (33)	1.54 (0.78 to 3.05)	2.10 (1.05 to 4.21)
**≥6 times**						
No social media use	7.7 (45)	0.63 (0.38 to 1.04)	0.71 (0.42 to 1.20)	12.8 (181)	1.01 (0.61 to 1.68)	1.01 (0.61 to 1.67)
1 - <30 mins	9.2 (89)	1.00	1.00	12.8 (29)	1.00	1.00
30 mins - <1 hr	11.4 (138)	1.53 (1.14 to 2.05)	1.62 (1.20 to 2.20)	9.7 (31)	0.95 (0.49 to 1.82)	0.97 (0.50 to 1.88)
1 - <2 hrs	13.3 (194)	2.28 (1.69 to 3.08)	2.61 (1.90 to 3.58)	13.6 (36)	1.49 (0.77 to 2.88)	1.74 (0.91 to 3.34)
≥2 hrs	17.0 (648)	3.26 (2.51 to 4.24)	4.80 (3.65 to 6.32)	14.2 (28)	1.47 (0.69 to 3.13)	2.18 (1.01 to 4.71)
	**Weighted prevalence % (observed n with outcome)**	**OR (95% CI)**	**AOR (95% CI)[a]**	**Weighted prevalence % (observed n with outcome)**	**OR (95% CI)**	**AOR (95% CI)[a]**
**B. Binge drinking (ref: no binge drinking**						
No social media use	28.7 (188)	0.51 (0.39 to 0.67)	0.54 (0.41 to 0.72)	53.1 (794)	0.81 (0.59 to 1.10)	0.75 (0.53 to 1.07)

1 - <30 mins	43.9 (433)	1.00	1.00	58.4 (131)	1.00	1.00
30 mins - <1 hr	53.1 (630)	1.44 (1.18 to 1.77)	1.51 (1.22 to 1.87)	53.8 (139)	0.83 (0.56 to 1.22)	0.79 (0.51 to 1.24)
1 - <2 hrs	60.5 (857)	1.96 (1.62 to 2.36)	2.06 (1.69 to 2.52)	57.0 (152)	0.94 (0.62 to 1.43)	1.03 (0.64 to 1.65)
≥2 hrs	66.7 (2,581)	2.55 (2.15 to 3.03)	3.07 (2.54 to 3.70)	59.1 (115)	1.03 (0.67 to 1.58)	1.19 (0.74 to 1.91)

Legend: Questionnaire imputed sample n = 8,987 (weighted sample: n = 6,175). Time-use-diary imputed sample: n = 2,520 (weighted sample: n = 5,005). [a] Adjusted for sex, ethnicity, religion, peer alcohol use, parental alcohol use, parental cigarette use, parental e-cigarette use, parenting style, previous cigarette use, previous e-cigarette use, anti-social behaviour, previous alcohol use, urbanicity, age, number of siblings in household, maternal age at participant birth, in-person activities, cognitive ability, mental health, risk-taking, and socioeconomic circumstances (family structure, household income, highest parental education in household, highest parental occupation in household, and area-level deprivation). Values may not add up due to rounding. Abbreviations: AOR = Adjusted odds ratio; ARRR = Adjusted relative risk ratio; CI = Confidence interval; Hr/s = Hour/s; Min/s = Minute/s; n = Number of participants; OR = Odds ratio; Ref = Reference category, and RRR = Relative risk ratio.

Table-G6. Time spent on social media on a normal weekday on risk of (A) frequency of alcohol use in the past month, and (B) binge drinking within the questionnaire imputed sample with additional adjustment for baseline outcome measures and previous social media use

	Questionnaire imputed sample (n = 8,987)				
	**Weighted prevalence % (observed n with outcome)**	**RRR (95% CI)**	**ARRR (95% CI)[a]**	**ARRR (95% CI)[b]**	**ARRR (95% CI)[c]**
**Time spent on social media on a normal weekday (questionnaire)**					
**A. Frequency of alcohol use in the past month (ref: never)**					
**1-2 times**					
No social media use	23.0 (160)	0.54 (0.41 to 0.72)	0.60 (0.44 to 0.80)	0.64 (0.47 to 0.86)	0.62 (0.46 to 0.84)
1 - <30 mins	31.6 (335)	1.00	1.00	1.00	1.00
30 mins - <1 hr	33.7 (404)	1.32 (1.06 to 1.64)	1.32 (1.05 to 1.67)	1.31 (1.03 to 1.66)	1.31 (1.04 to 1.65)
1 - <2 hrs	35.9 (515)	1.79 (1.44 to 2.22)	1.80 (1.43 to 2.26)	1.71 (1.36 to 2.16)	1.77 (1.41 to 2.22)
≥2 hrs	35.0 (1,391)	1.96 (1.65 to 2.32)	2.10 (1.73 to 2.55)	1.80 (1.48 to 2.20)	2.03 (1.67 to 2.46)
**3-5 times**					
No social media use	10.0 (35)	0.48 (0.32 to 0.73)	0.55 (0.36 to 0.85)	0.61 (0.39 to 0.96)	0.57 (0.37 to 0.88)
1 - <30 mins	15.7 (89)	1.00	1.00	1.00	1.00
30 mins - <1 hr	19.2 (151)	1.59 (1.18 to 2.13)	1.69 (1.24 to 2.30)	1.60 (1.17 to 2.1	1.66 (1.22 to 2.26)
1 - <2 hrs	22.8 (204)	2.42 (1.86 to 3.15)	2.63 (1.98 to 3.50)	2.34 (1.75 to 3.12)	2.59 (1.94 to 3.45)
≥2 hrs	24.1 (584)	2.75 (2.18 to 3.48)	3.45 (2.68 to 4.45)	2.54 (1.96 to 3.29)	3.34 (2.59 to 4.30)
**≥6 times**					
No social media use	8.5 (28)	0.63 (0.38 to 1.04)	0.71 (0.42 to 1.20)	0.84 (0.49 to 1.42)	0.76 (0.45 to 1.29)
1 - <30 mins	10.8 (61)	1.00	1.00	1.00	1.00
30 mins - <1 hr	12.9 (98)	1.53 (1.14 to 2.05)	1.62 (1.20 to 2.20)	1.47 (1.07 to 2.01)	1.58 (1.17 to 2.13)
1 - <2 hrs	14.9 (132)	2.28 (1.69 to 3.08)	2.61 (1.90 to 3.58)	2.11 (1.52 to 2.93)	2.53 (1.84 to 3.47)
≥2 hrs	17.3 (414)	3.26 (2.51 to 4.24)	4.80 (3.65 to 6.32)	2.86 (2.12 to 3.87)	4.43 (3.36 to 5.82)
	**Weighted prevalence % (observed n with outcome)**	**OR (95% CI)**	**AOR (95% CI)[a]**	**AOR (95% CI)[b]**	**AOR (95% CI)[c]**
**B. Binge drinking (ref: no binge drinking)**					
No social media use	28.7 (188)	0.51 (0.39 to 0.67)	0.54 (0.41 to 0.72)	0.59 (0.44 to 0.79)	0.57 (0.42 to 0.76)
1 - <30 mins	43.9 (433)	1.00	1.00	1.00	1.00

30 mins - <1 hr	53.1 (630)	1.44 (1.18 to 1.77)	1.51 (1.22 to 1.87)	1.43 (1.15 to 1.79)	1.48 (1.20 to 1.83)
1 - <2 hrs	60.5 (857)	1.96 (1.62 to 2.36)	2.06 (1.69 to 2.52)	1.81 (1.46 to 2.23)	2.01 (1.64 to 2.46)
≥2 hrs	66.7 (2,581)	2.55 (2.15 to 3.03)	3.07 (2.54 to 3.70)	2.17 (1.78 to 2.65)	2.91 (2.41 to 3.51)

Legend: Questionnaire imputed sample: n = 8,987 (weighted sample: n = 6,175). [a] Adjusted for sex, ethnicity, religion, peer alcohol use, parental alcohol use, parental cigarette use, parental e-cigarette use, parenting style, previous cigarette use, previous e-cigarette use, anti-social behaviour, previous alcohol use, urbanicity, age, number of siblings in household, maternal age at participant birth, in-person activities, cognitive ability, mental health, risk-taking, and socioeconomic circumstances (family structure, household income, highest parental education in household, highest parental occupation in household, and area-level deprivation). [b] Additional adjustment for baseline binge drinking (age 14-years) and baseline frequency of alcohol use in the last year (age 14-years). [c] Additional adjustment for previous social media use (age 11-years).Values may not add up due to rounding. Abbreviations: AOR = Adjusted odds ratio; ARRR = Adjusted relative risk ratio; CI = Confidence interval; Hr/s = Hour/s; Min/s = Minute/s; n = Number of participants; OR = Odds ratio; Ref = Reference category, and RRR = Relative risk ratio.

Table-G7. Average time spent on social media across a normal weekday and weekend day on risk of (A) frequency of alcohol use in the past month, and (B) binge drinking within the time-use-diary imputed sample with additional adjustment for baseline outcome measures and previous social media use

	Time-use-diary imputed sample (n = 2,520)				
	**Weighted prevalence % (observed n with outcome)**	**RRR (95% CI)**	**ARRR (95% CI)[a]**	**ARRR (95% CI)[b]**	**ARRR (95% CI)[c]**
**A. Frequency of alcohol use in the past month (ref: never)**					
**1-2 times**					
No social media use	31.7 (367)	0.80 (0.59 to 1.09)	0.88 (0.64 to 1.20)	0.82 (0.60 to 1.12)	0.89 (0.65 to 1.21)
1 - <30 mins	37.2 (183)	1.00	1.00	1.00	1.00
30 mins - <1 hr	38.2 (149)	1.42 (0.93 to 2.17)	1.45 (0.97 to 2.17)	1.31 (0.87 to 1.96)	1.43 (0.96 to 2.15)
1 - <2 hrs	31.9 (85)	1.03 (0.64 to 1.64)	1.02 (0.62 to 1.67)	0.93 (0.57 to 1.50)	1.04 (0.64 to 1.69)
≥2 hrs	39.0 (65)	1.26 (0.78 to 2.03)	1.48 (0.91 to 2.40)	1.23 (0.75 to 2.02)	1.40 (0.87 to 2.30)
**3-5 times**					
No social media use	17.7 (210)	1.00 (0.69 to 1.45)	1.13 (0.75 to 1.71)	1.01 (0.66 to 1.55)	1.12 (0.74 to 1.69)
1 - <30 mins	16.7 (88)	1.00	1.00	1.00	1.00
30 mins - <1 hr	20.3 (79)	1.69 (1.09 to 2.60)	1.80 (1.14 to 2.82)	1.53 (0.97 to 2.40)	1.71 (1.09 to 2.68)
1 - <2 hrs	22.2 (63)	1.60 (0.95 to 2.68)	1.71 (0.98 to 2.96)	1.37 (0.78 to 2.40)	1.69 (0.97 to 2.94)
≥2 hrs	20.0 (33)	1.44 (0.76 to 2.75)	1.94 (0.98 to 3.83)	1.39 (0.71 to 2.70)	1.72 (0.89 to 3.35)
**≥6 times**					
No social media use	12.4 (140)	1.18 (0.75 to 1.84)	1.15 (0.71 to 1.86)	1.01 (0.62 to 1.66)	1.12 (0.69 to 1.79)
1 - <30 mins	9.9 (50)	1.00	1.00	1.00	1.00
30 mins - <1 hr	15.5 (53)	2.15 (1.25 to 3.68)	2.50 (1.47 to 4.25)	2.10 (1.21 to 3.62)	2.29 (1.37 to 3.84)
1 - <2 hrs	15.9 (39)	1.91 (1.04 to 3.48)	2.22 (1.17 to 4.20)	1.57 (0.82 to 3.00)	2.17 (1.13 to 4.16)
≥2 hrs	11.1 (22)	1.33 (0.67 to 2.65)	2.15 (1.03 to 4.51)	1.38 (0.63 to 2.00)	1.77 (0.83 to 3.80)
	**Weighted prevalence % (observed n with outcome)**	**OR (95% CI)**	**AOR (95% CI)[a]**	**AOR (95% CI)[b]**	**AOR (95% CI)[c]**
**B. Binge drinking (ref: no binge drinking)**					
No social media use	50.8 (581)	0.77 (0.60 to 1.00)	0.73 (0.55 to 0.96)	0.62 (0.46 to 0.84)	0.74 (0.56 to 0.97)
1 - <30 mins	57.2 (270)	1.00	1.00	1.00	1.00

30 mins - <1 hr	58.3 (218)	1.05 (0.78 to 1.42)	1.10 (0.80 to 1.52)	0.89 (0.64 to 1.24)	1.05 (0.77 to 1.45)
1 - <2 hrs	59.6 (162)	1.10 (0.76 to 1.61)	1.13 (0.75 to 1.70)	0.88 (0.57 to 1.36)	1.13 (0.75 to 1.69)
≥2 hrs	57.1 (99)	1.00 (0.66 to 1.51)	1.33 (0.87 to 2.05)	0.91 (0.58 to 1.44)	1.18 (0.77 to 1.81)

Legend: Time-use-diary imputed sample: n = 2,520 (weighted sample: n = 5,005). [a] Adjusted for sex, ethnicity, religion, peer alcohol use, parental alcohol use, parental cigarette use, parental e-cigarette use, parenting style, previous cigarette use, previous e-cigarette use, anti-social behaviour, previous alcohol use, urbanicity, age, number of siblings in household, maternal age at participant birth, in-person activities, cognitive ability, mental health, risk-taking, and socioeconomic circumstances (family structure, household income, highest parental education in household, highest parental occupation in household, and area-level deprivation). [b] Additional adjustment for baseline binge drinking (age 14-years) and baseline frequency of alcohol use in the last year (age 14-years). [c] Additional adjustment for previous social media use (age 11-years).Values may not add up due to rounding. Abbreviations: AOR = Adjusted odds ratio; ARRR = Adjusted relative risk ratio; CI = Confidence interval; Hr/s = Hour/s; Min/s = Minute/s; n = Number of participants; OR = Odds ratio; Ref = Reference category, and RRR = Relative risk ratio.

Table-G8. Time spent on social media on a normal weekday on risk of (A) frequency of alcohol use in the past month, and (B) binge drinking within the questionnaire imputed sample replacing '1-<30 minutes' reference category with 'no social media use'

	Questionnaire imputed sample (n = 8,987)		
	**Weighted prevalence % (observed n with outcome)**	**RRR (95% CI)**	**ARRR (95% CI)[a]**
**Time spent on social media on a normal weekday (questionnaire)**			
**A. Frequency of alcohol use in the past month (ref: never)**			
**1-2 times**			
No social media use	23.0 (160)	1.00	1.00
1 - <30 mins	31.6 (335)	1.84 (1.39 to 2.43)	1.68 (1.25 to 2.25)
30 mins - <1 hr	33.7 (404)	2.42 (1.83 to 3.20)	2.22 (1.66 to 2.96)
1 - <2 hrs	35.9 (515)	3.29 (2.50 to 4.31)	3.02 (2.28 to 3.99)
≥2 hrs	35.0 (1,391)	3.59 (2.83 to 4.56)	3.52 (2.70 to 4.59)
**3-5 times**			
No social media use	10.0 (35)	1.00	1.00
1 - <30 mins	15.7 (89)	2.07 (1.37 to 3.13)	1.82 (1.18 to 2.81)
30 mins - <1 hr	19.2 (151)	3.29 (2.17 to 4.97)	3.07 (1.98 to 4.75)
1 - <2 hrs	22.8 (204)	5.01 (3.37 to 7.45)	4.79 (3.14 to 7.31)
≥2 hrs	24.1 (584)	5.69 (3.96 to 8.18)	6.28 (4.22 to 9.35)
**≥6 times**			
No social media use	8.5 (28)	1.00	1.00
1 - <30 mins	10.8 (61)	1.59 (0.96 to 2.62)	1.41 (0.84 to 2.37)
30 mins - <1 hr	12.9 (98)	2.43 (1.53 to 3.86)	2.28 (1.39 to 3.75)
1 - <2 hrs	14.9 (132)	3.63 (2.32 to 5.68)	3.67 (2.26 to 5.96)
≥2 hrs	17.3 (414)	5.18 (3.40 to 7.89)	6.75 (4.28 to 10.67)
	**Weighted prevalence % (observed n with outcome)**	**OR (95% CI)**	**AOR (95% CI)[a]**
**B. Binge drinking (ref: no binge drinking)**			
No social media use	28.7 (188)	1.00	1.00
1 - <30 mins	43.9 (433)	1.95 (1.50 to 2.55)	1.85 (1.38 to 2.46)
30 mins - <1 hr	53.1 (630)	2.82 (2.19 to 3.63)	2.79 (2.11 to 3.69)
1 - <2 hrs	60.5 (857)	3.82 (2.99 to 4.88)	3.81 (2.88 to 5.02)
≥2 hrs	66.7 (2,581)	4.98 (3.97 to 6.26)	5.66 (4.34 to 7.37)

Legend: Questionnaire imputed sample: n = 8,987 (weighted sample: n = 6,175). [a] Adjusted for sex, ethnicity, religion, peer alcohol use, parental alcohol use, parental cigarette use, parental e-cigarette use, parenting style, previous cigarette use, previous e-cigarette use, anti-social behaviour, previous alcohol use, urbanicity, age, number of siblings in household, maternal age at participant birth, in-person activities, cognitive ability, mental health, risk-taking, and socioeconomic circumstances (family structure, household income, highest parental education in household, highest parental occupation in household, and area-level deprivation). Values may not add up due to rounding. Abbreviations: AOR = Adjusted odds ratio; ARRR = Adjusted relative risk ratio; CI = Confidence interval; Hr/s = Hour/s; Min/s = Minute/s; n = Number of participants; OR = Odds ratio; Ref = Reference category, and RRR = Relative risk ratio.

APPENDIX-H. Differential effect of social media on binge drinking by socioeconomic circumstance

Assessment on the additive scale using risk differences (RDs)

Table-H1. Participant binge drinking according to time spent on social media, within strata of parental education and according to 'combinations' of time spent on social media and parental education within the questionnaire and time-use-diary imputed samples (condensed table shown in manuscript)

	**Questionnaire imputed sample (n = 8,954)**		**Time-use-diary imputed sample (n = 2,520)**	
	**High parental education**	**Low parental education**	**High parental education**	**Low parental education**
**Outcome: Binge drinking**				
**Weighted prevalence % (observed n with outcome/without outcome)**				
No social media use	28.1 (124/310)	31.5 (64/232)	53.3 (408/407)	47.9 (172/191)
1 - <30 mins	46.4 (296/415)	36.6 (135/295)	60.8 (201/153)	51.6 (69/75)
30 mins - <1 hr	57.1 (430/394)	47.6 (198/303)	63.1 (157/108)	49.7 (61/64)
1 - <2 hrs	63.4 (575/364)	52.7 (278/341)	61.1 (107/67)	51.6 (54/48)
≥2 hrs	69.2 (1,478/774)	61.6 (1,092/857)	52.5 (58/46)	57.3 (41/34)
**Unadjusted RD (95% CI; *p-values*) for time spent on social media within strata of parental education**				
No social media use	Ref	Ref	Ref	Ref
1 - <30 mins	18.3 (11.1 to 25.4; <0.0001)	5.1 (-8.1 to 18.3; 0.45)	7.4 (0.5 to 14.4; 0.037)	3.6 (-10.7 to 18.0; 0.62)
30 mins - <1 hr	28.9 (22.3 to 35.6; <0.0001)	16.1 (4.0 to 28.1; 0.009)	9.7 (2.7 to 16.8; 0.007)	1.8 (-12.5 to 16.2; 0.80)
1 - <2 hrs	35.3 (29.5 to 41.0; <0.0001)	21.2 (9.0 to 33.5; 0.001)	7.8 (-5.4 to 21.0; 0.25)	3.7 (-10.1 to 17.4; 0.60)
≥2 hrs	41.0 (35.8 to 46.3; <0.0001)	30.1 (19.2 to 41.0; <0.0001)	-0.9 (-14.1 to 12.3; 0.90)	9.4 (-8.8 to 27.6; 0.31)
**Unadjusted RD (95% CI; *p-value*) for time spent on social media and parental education**				
No social media use	-3.4 (-14.9 to 8.2; 0.57)	Ref	5.4 (-3.2 to 14.1; 0.22)	Ref
1 - <30 mins	14.9 (3.0 to 26.9; 0.014)	5.1 (-8.1 to 18.3; 0.45)	12.8 (3.7 to 22.0; 0.006)	3.6 (-10.7 to 18.0; 0.62)
No social media use	-3.4 (-14.9 to 8.2; 0.57)	Ref	5.4 (-3.2 to 14.1; 0.22)	Ref
30 mins - <1 hr	25.6 (14.1 to 37.1; <0.0001)	16.1 (4.0 to 28.1; 0.009)	15.2 (5.6 to 24.8; 0.002)	1.8 (-12.5 to 16.2; 0.80)
No social media use	-3.4 (-14.9 to 8.2; 0.57)	Ref	5.4 (-3.2 to 14.1; 0.22)	Ref
1 - <2 hrs	31.9 (20.9 to 42.9; <0.0001)	21.2 (9.0 to 33.5; 0.001)	13.2 (-0.9 to 27.4; 0.067)	3.7 (-10.1 to 17.4; 0.60)
No social media use	-3.4 (-14.9 to 8.2; 0.57)	Ref	5.4 (-3.2 to 14.1; 0.22)	Ref
≥2 hrs	37.7 (26.7 to 48.7; <0.0001)	30.1 (19.2 to 41.0; <0.0001)	4.5 (-8.9 to 18.0; 0.51)	9.4 (-8.8 to 27.6; 0.31)
**Unadjusted measure of additive effect modification[a] and interaction[b] (95% CI; *p-value*)**				
No social media use	Ref		Ref	

1 - <30 mins	13.2 (-1.3 to 27.7; 0.074)		3.8 (-11.9 to 19.4; 0.64)	
30 mins - <1 hr	12.8 (-0.8 to 26.5; 0.064)		7.9 (-7.6 to 23.4; 0.32)	
1 - <2 hrs	14.0 (0.7 to 27.4; 0.040)		4.1 (-16.3 to 24.6; 0.69)	
≥2 hrs	10.9 (-1.0 to 22.9; 0.073)		-10.3 (-32.0 to 11.4; 0.35)	
**Adjusted[c] RD (95% CI; *p-value*) for time spent on social media within strata of parental education**				
No social media use	Ref	Ref	Ref	Ref
1 - <30 mins	15.2 (8.3 to 22.1; <0.0001)	3.4 (-7.8 to 14.7; 0.55)	7.4 (0.8 to 14.0; 0.029)	2.4 (-9.7 to 14.4; 0.70)
30 mins - <1 hr	27.4 (21.2 to 33.7; <0.0001)	12.1 (1.1 to 23.1; 0.031)	10.0 (3.1 to 16.9; 0.005)	1.9 (-11.2 to 15.1; 0.77)
1 - <2 hrs	33.0 (26.9 to 39.2; <0.0001)	15.6 (4.7 to 26.4; 0.005)	8.4 (-4.4 to 21.2; 0.20)	4.6 (-8.8 to 18.0; 0.50)
≥2 hrs	40.0 (34.7 to 45.3; <0.0001)	23.8 (13.6 to 34.0; <0.0001)	6.4 (-5.9 to 18.8; 0.31)	11.7 (-2.8 to 26.2; 0.11)
**Adjusted[c] RD (95% CI; *p-value*) for time spent on social media and parental education**				
No social media use	-9.5 (-19.1 to 0.2; 0.055)	Ref	1.2 (-7.1 to 9.6; 0.77)	Ref
1 - <30 mins	5.8 (-4.5 to 16.0; 0.27)	3.4 (-7.8 to 14.7; 0.55)	8.6 (-0.4 to 17.5; 0.060)	2.4 (-9.7 to 14.4; 0.70)
No social media use	-11.3 (-20.8 to -1.8; 0.020)	Ref	1.3 (-6.6 to 9.4; 0.74)	Ref
30 mins - <1 hr	16.1 (6.4 to 25.8; 0.001)	12.1 (1.1 to 23.1; 0.031)	11.3 (1.8 to 20.8; 0.020)	1.9 (-11.2 to 15.1; 0.77)
No social media use	-8.9 (-18.6 to 0.8; 0.073)	Ref	0.8 (-7.7 to 9.3; 0.85)	Ref
1 - <2 hrs	24.2 (14.4 to 33.9; <0.0001)	15.6 (4.7 to 26.4; 0.005)	9.2 (-5.9 to 24.4; 0.23)	4.6 (-8.8 to 18.0; 0.50)
No social media use	-12.2 (-22.4 to -1.9; 0.020)	Ref	0.8 (-7.4 to 9.0; 0.85)	Ref
≥2 hrs	27.8 (17.8 to 37.8; <0.0001)	23.8 (13.6 to 34.0; <0.0001)	7.2 (-5.3 to 19.8; 0.26)	11.7 (-2.8 to 26.2; 0.11)
**Adjusted[c] measure of additive effect modification[a] and interaction[b] (95% CI; *p-value*)**				
No social media use	Ref		Ref	
1 - <30 mins	11.8 (-0.6 to 24.2; 0.063)		5.0 (-8.6 to 18.6; 0.47)	
30 mins - <1 hr	15.3 (3.2 to 27.5; 0.014)		8.0 (-6.4 to 22.5; 0.27)	
1 - <2 hrs	17.5 (5.6 to 29.3; 0.004)		3.8 (-14.8 to 22.5; 0.69)	
≥2 hrs	16.2 (5.2 to 27.2; 0.004)		-5.3 (-22.7 to 12.1; 0.55)	

Legend: Questionnaire imputed sample: n = 8,954 (weighted sample: n = 6,976). Time-use-diary imputed sample: n = 2,520 (weighted sample: n = 5,727). [a] Measure of effect modification on an additive scale represents the size of the absolute difference between the RDs for participant binge drinking by time spent on social media, within the high parental education group compared with baseline (low parental education group).[b] Measure of interaction on an additive scale represents the size of the difference between the RD in participants with: for example, high parental education and 1-<30 minutes social media use, compared with the RD for participants with high parental education and no social media use, plus the RD for those with low parental education and 1-<30 minutes social media use. [c] Adjusted for sex, ethnicity, religion, peer alcohol use, parental alcohol use, parental cigarette use, parental e-cigarette use, parenting style, previous cigarette use, previous e-cigarette use, anti-social behaviour, previous alcohol use, urbanicity, age, number of siblings in household, maternal age at participant birth, in-person activities, cognitive ability, mental health, and risk-taking. Values may not add up due to rounding. Abbreviations: CI = Confidence interval; Hr/s = Hour/s; Min/s = Minute/s; n = Number of participants; RD = Risk differences, and Ref = Reference category.

Table-H2. Participant binge drinking according to time spent on social media, within strata of parental education and according to 'combinations' of time spent on social media and parental education within the questionnaire and time-use-diary complete case samples

	**Questionnaire complete case sample (n = 5,317)**		**Time-use-diary complete case sample (n = 1,826)**	
	**High parental education**	**Low parental education**	**High parental education**	**Low parental education**
**Outcome: Binge drinking**				
**Weighted prevalence % (observed n with outcome/without outcome)**				
No social media use	29.3 (78/188)	35.4 (31/85)	54.2 (296/305)	54.7 (119/111)
1 - <30 mins	49.6 (205/255)	39.7 (79/132)	65.0 (159/104)	51.3 (44/48)
30 mins - <1 hr	59.3 (307/243)	54.5 (123/138)	61.5 (126/83)	44.2 (40/45)
1 - <2 hrs	64.4 (387/213)	55.5 (162/163)	56.7 (82/51)	61.6 (41/26)
≥2 hrs	71.0 (991/480)	65.5 (642/415)	48.5 (45/38)	57.3 (34/29)
**Unadjusted RD (95% CI; *p-value*) for time spent on social media within strata of parental education**				
No social media use	Ref	Ref	Ref	Ref
1 - <30 mins	20.3 (11.3 to 29.3; <0.0001)	4.3 (-10.2 to 18.8; 0.56)	10.8 (2.5 to 19.2; 0.011)	-3.4 (-21.2 to 14.4; 0.71)
30 mins - <1 hr	30.1 (21.9 to 38.2; <0.0001)	19.1 (4.6 to 33.7; 0.010)	7.3 (-1.1 to 15.8; 0.090)	-10.5 (-25.8 to 4.9; 0.18)
1 - <2 hrs	35.2 (27.9 to 42.5; <0.0001)	20.1 (5.1 to 35.1; 0.009)	2.5 (-14.4 to 19.5; 0.77)	7.0 (-10.5 to 24.4; 0.43)
≥2 hrs	41.7 (34.8 to 48.6; <0.0001)	30.1 (17.1 to 43.2; <0.0001)	-5.7 (-20.1 to 8.7; 0.44)	2.6 (-17.5 to 22.7; 0.80)
**Unadjusted RD (95% CI; *p-value*) for time spent on social media and parental education**				
No social media use	-6.1 (-19.6 to 7.4; 0.38)	Ref	-0.5 (-11.7 to 10.7; 0.93)	Ref
1 - <30 mins	14.2 (0.7 to 27.7; 0.039)	4.3 (-10.2 to 18.8; 0.56)	10.3 (-1.2 to 21.8; 0.078)	-3.4 (-21.2 to 14.4; 0.71)
No social media use	-6.1 (-19.6 to 7.4; 0.38)	Ref	-0.5 (-11.7 to 10.7; 0.93)	Ref
30 mins - <1 hr	24.0 (10.7 to 37.2; <0.0001)	19.1 (4.6 to 33.7; 0.010)	6.8 (-5.3 to 18.9; 0.27)	-10.5 (-25.8 to 4.9; 0.18)
No social media use	-6.1 (-19.6 to 7.4; 0.38)	Ref	-0.5 (-11.7 to 10.7; 0.93)	Ref
1 - <2 hrs	29.1 (16.0 to 42.1; <0.0001)	20.1 (5.1 to 35.1; 0.009)	2.0 (-16.7 to 20.8; 0.83)	7.0 (-10.5 to 24.4; 0.43)
No social media use	-6.1 (-19.6 to 7.4; 0.38)	Ref	-0.5 (-11.7 to 10.7; 0.93)	Ref
≥2 hrs	35.6 (23.1 to 48.2; <0.0001)	30.1 (17.1 to 43.2; <0.0001)	-6.2 (-21.2 to 8.9; 0.42)	2.6 (-17.5 to 22.7; 0.80)
**Unadjusted measure of additive effect modification[a] and interaction[b] (95% CI; *p-value*)**				
No social media use	Ref		Ref	
1 - <30 mins	16.0 (-1.3 to 33.3; 0.069)		14.2 (-5.3 to 33.8; 0.15)	
30 mins - <1 hr	10.9 (-5.0 to 26.8; 0.18)		17.8 (1.1 to 34.4; 0.036)	
1 - <2 hrs	15.1 (-1.1 to 31.2; 0.067)		-4.4 (-29.2 to 20.3; 0.73)	
≥2 hrs	11.6 (-2.8 to 26.0; 0.12)		-8.3 (-33.1 to 16.5; 0.51)	

**Adjusted[c] RD (95% CI; *p-value*) for time spent on social media within strata of parental education**				
No social media use	Ref	Ref	Ref	Ref
1 - <30 mins	18.4 (10.4 to 26.4; <0.0001)	4.3 (-7.3 to 15.8; 0.47)	9.9 (1.8 to 17.9; 0.016)	-5.5 (-19.1 to 8.2; 0.43)
30 mins - <1 hr	29.0 (21.3 to 36.8; <0.0001)	16.4 (4.1 to 28.8; 0.009)	9.7 (2.0 to 17.4; 0.013)	-6.1 (-20.6 to 8.5; 0.42)
1 - <2 hrs	34.4 (26.9 to 41.9; <0.0001)	14.8 (2.0 to 27.6; 0.023)	2.9 (-11.7 to 17.6; 0.69)	7.9 (-7.8 to 23.6; 0.32)
≥2 hrs	41.9 (35.2 to 48.6; <0.0001)	27.5 (16.0 to 39.0; <0.0001)	1.4 (-12.8 to 15.6; 0.85)	4.9 (-10.8 to 20.5; 0.54)
**Adjusted[c] RD (95% CI; *p-value*) for time spent on social media and parental education**				
No social media use	-11.9 (-22.9 to -0.8; 0.036)	Ref	-2.1 (-12.7 to 8.4; 0.69)	Ref
1 - <30 mins	6.5 (-4.7 to 17.6; 0.25)	4.3 (-7.3 to 15.8; 0.47)	7.7 (-2.9 to 18.4; 0.15)	-5.5 (-19.1 to 8.2; 0.43)
No social media use	-12.2 (-23.7 to -0.8; 0.037)	Ref	-1.7 (-11.9 to 8.5; 0.75)	Ref
30 mins - <1 hr	16.8 (5.7 to 27.9; 0.003)	16.4 (4.1 to 28.8; 0.009)	8.0 (-3.2 to 19.2; 0.16)	-6.1 (-20.6 to 8.5; 0.42)
No social media use	-10.8 (-21.4 to -0.1; 0.047)	Ref	-1.4 (-12.0 to 9.2; 0.79)	Ref
1 - <2 hrs	23.6 (12.6 to 34.7; <0.0001)	14.8 (2.0 to 27.6; 0.023)	1.5 (-15.3 to 18.4; 0.86)	7.9 (-7.8 to 23.6; 0.32)
No social media use	-12.6 (-24.1 to -1.1; 0.032)	Ref	-2.0 (-12.5 to 8.5; 0.71)	Ref
≥2 hrs	29.3 (18.6 to 40.0; <0.0001)	27.5 (16.0 to 39.0; <0.0001)	-0.6 (-14.3 to 13.2; 0.94)	4.9 (-10.8 to 20.5; 0.54)
**Adjusted[c] measure of additive effect modification[a] and interaction[b] (95% CI; *p-value*)**				
No social media use	Ref		Ref	
1 - <30 mins	14.1 (-0.0 to 28.2; 0.050)		15.3 (-0.3 to 30.9; 0.054)	
30 mins - <1 hr	12.6 (-0.9 to 26.1; 0.068)		15.8 (-0.5 to 32.0; 0.058)	
1 - <2 hrs	19.6 (6.4 to 32.8; 0.004)		-4.9 (-25.9 to 16.1; 0.64)	
≥2 hrs	14.4 (1.7 to 27.1; 0.026)		-3.5 (-23.9 to 17.0; 0.74)	

Legend: Questionnaire complete case sample: n = 5,317 (weighted sample: n = 4,095). Time-use-diary complete case sample: n = 1,826 (weighted sample: n = 3,902). [a] Measure of effect modification on an additive scale represents the size of the absolute difference between the RDs for participant binge drinking by time spent on social media, within the high parental education group compared with baseline (low parental education group).[b] Measure of interaction on an additive scale represents the size of the difference between the RD in participants with: for example, high parental education and 1-<30 minutes social media use, compared with the RD for participants with high parental education and no social media use, plus the RD for those with low parental education and 1-<30 minutes social media use. [c] Adjusted for sex, ethnicity, religion, peer alcohol use, parental alcohol use, parental cigarette use, parental e-cigarette use, parenting style, previous cigarette use, previous e-cigarette use, anti-social behaviour, previous alcohol use, urbanicity, age, number of siblings in household, maternal age at participant birth, in-person activities, cognitive ability, mental health, and risk-taking. Values may not add up due to rounding. Abbreviations: CI = Confidence interval; Hr/s = Hour/s; Min/s = Minute/s; n = Number of participants; RD = Risk differences, and Ref = Reference category.

Assessment on the multiplicative scale using risk ratios (RRs)

Table-H3. Participant binge drinking according to time spent on social media, within strata of parental education and according to 'combinations' of time spent on social media and parental education within the questionnaire and time-use-diary imputed samples

	**Questionnaire imputed sample (n = 8,954)**		**Time-use-diary imputed sample (n = 2,520)**	
	**High parental education**	**Low parental education**	**High parental education**	**Low parental education**
**Outcome: Binge drinking**				
**Weighted prevalence % (observed n with outcome/without outcome)**				
No social media use	28.1 (124/310)	31.5 (64/232)	53.3 (408/407)	47.9 (172/191)
1 - <30 mins	46.4 (296/415)	36.6 (135/295)	60.8 (201/153)	51.6 (69/75)
30 mins - <1 hr	57.1 (430/394)	47.6 (198/303)	63.1 (157/108)	49.7 (61/64)
1 - <2 hrs	63.4 (575/364)	52.7 (278/341)	61.1 (107/67)	51.6 (54/48)
≥2 hrs	69.2 (1,478/774)	61.6 (1,092/857)	52.5 (58/46)	57.3 (41/34)
**Unadjusted RR (95% CI; *p-value*) for time spent on social media within strata of parental education**				
No social media use	Ref	Ref	Ref	Ref
1 - <30 mins	1.65 (1.34 to 2.03; <0.0001)	1.16 (0.78 to 1.74; 0.45)	1.14 (1.01 to 1.29; 0.035)	1.08 (0.81 to 1.43; 0.61)
30 mins - <1 hr	2.03 (1.67 to 2.46; <0.0001)	1.51 (1.06 to 2.16; 0.023)	1.18 (1.05 to 1.33; 0.006)	1.04 (0.78 to 1.39; 0.80)
1 - <2 hrs	2.25 (1.89 to 2.69; <0.0001)	1.68 (1.18 to 2.40; 0.005)	1.15 (0.92 to 1.43; 0.22)	1.08 (0.82 to 1.41; 0.60)
≥2 hrs	2.46 (2.07 to 2.92; <0.0001)	1.96 (1.40 to 2.75; <0.0001)	0.98 (0.49 to 0.58; <0.0001)	1.20 (0.86 to 1.66; 0.28)
**Unadjusted RR (95% CI; *p-value*) for time spent on social media and parental education**				
No social media use	0.90 (0.62 to 1.30; 0.56)	Ref	1.11 (0.93 to 1.33; 0.23)	Ref
1 - <30 mins	1.48 (1.03 to 2.11; 0.033)	1.16 (0.78 to 1.74; 0.45)	1.27 (1.06 to 1.51; 0.009)	1.08 (0.81 to 1.43; 0.61)
No social media use	0.90 (0.62 to 1.30; 0.56)	Ref	1.11 (0.93 to 1.33; 0.23)	Ref
30 mins - <1 hr	1.82 (1.28 to 2.57; 0.001)	1.51 (1.06 to 2.16; 0.023)	1.32 (1.10 to 1.58; 0.003)	1.04 (0.78 to 1.39; 0.80)
No social media use	0.90 (0.62 to 1.30; 0.56)	Ref	1.11 (0.93 to 1.33; 0.23)	Ref
1 - <2 hrs	2.02 (1.44 to 2.84; <0.0001)	1.68 (1.18 to 2.40; 0.005)	1.28 (1.00 to 1.63; 0.051)	1.08 (0.82 to 1.41; 0.60)
No social media use	0.90 (0.62 to 1.30; 0.56)	Ref	1.11 (0.93 to 1.33; 0.23)	Ref
≥2 hrs	2.20 (1.57 to 3.10; <0.0001)	1.96 (1.40 to 2.75; <0.0001)	1.09 (0.84 to 1.42; 0.50)	1.20 (0.86 to 1.66; 0.28)
**Unadjusted measure of multiplicative effect modification and interaction (95% CI; *p-value*)**				
No social media use	Ref		Ref	
1 - <30 mins	1.42 (0.92 to 2.19; 0.16)		1.06 (0.78 to 1.43; 0.71)	
30 mins - <1 hr	1.34 (0.90 to 1.99; 0.15)		1.14 (0.84 to 1.55; 0.41)	

1 - <2 hrs	1.34 (0.91 to 1.98; 0.14)		1.06 (0.73 to 1.55; 0.74)	
≥2 hrs	1.25 (0.86 to 1.82; 0.23)		0.82 (0.55 to 1.23; 0.34)	
**Adjusted[a] RR (95% CI; *p-value*) for time spent on social media within strata of parental education**				
No social media use	Ref	Ref	Ref	Ref
1 - <30 mins	1.51 (1.23 to 1.85; <0.0001)	1.11 (0.79 to 1.57; 0.54)	1.13 (1.01 to 1.27; 0.037)	1.04 (0.81 to 1.33; 0.75)
30 mins - <1 hr	1.93 (1.60 to 2.32; <0.0001)	1.37 (0.99 to 1.90; 0.054)	1.20 (1.06 to 1.35; 0.003)	1.05 (0.80 to 1.37; 0.74)
1 - <2 hrs	2.13 (1.77 to 2.57; <0.0001)	1.49 (1.09 to 2.06; 0.014)	1.17 (0.94 to 1.45; 0.15)	1.09 (0.84 to 1.41; 0.53)
≥2 hrs	2.42 (2.04 to 2.86; <0.0001)	1.77 (1.29 to 2.43; 0.001)	1.15 (0.90 to 1.47; 0.25)	1.29 (1.00 to 1.68; 0.054)
**Adjusted[a] RR (95% CI; *p-value*) for time spent on social media and parental education**				
No social media use	0.77 (0.56 to 1.05; 0.095)	Ref	1.03 (0.87 to 1.22; 0.740)	Ref
1 - <30 mins	1.16 (0.85 to 1.56; 0.35)	1.11 (0.79 to 1.57; 0.54)	1.16 (0.98 to 1.38; 0.081)	1.04 (0.81 to 1.33; 0.75)
No social media use	0.76 (0.56 to 1.03; 0.080)	Ref	1.03 (0.87 to 1.21; 0.75)	Ref
30 mins - <1 hr	1.46 (1.10 to 1.95; 0.010)	1.37 (0.99 to 1.90; 0.054)	1.23 (1.02 to 1.48; 0.028)	1.05 (0.80 to 1.37; 0.74)
No social media use	0.80 (0.58 to 1.11; 0.18)	Ref	1.02 (0.86 to 1.21; 0.83)	Ref
1 - <2 hrs	1.72 (1.28 to 2.31; <0.0001)	1.49 (1.09 to 2.06; 0.014)	1.19 (0.91 to 1.56; 0.20)	1.09 (0.84 to 1.41; 0.53)
No social media use	0.77 (0.55 to 1.09; 0.14)	Ref	1.01 (0.86 to 1.20; 0.88)	Ref
≥2 hrs	1.86 (1.36 to 2.55; <0.0001)	1.77 (1.29 to 2.43; 0.001)	1.17 (0.90 to 1.50; 0.23)	1.29 (1.00 to 1.68; 0.054)
**Adjusted[a] measure of multiplicative effect modification and interaction (95% CI; *p-value*)**				
No social media use	Ref		Ref	
1 - <30 mins	1.35 (0.93 to 1.97; 0.11)		1.09 (0.83 to 1.42; 0.55)	
30 mins - <1 hr	1.40 (0.98 to 2.01; 0.063)		1.14 (0.86 to 1.53; 0.36)	
1 - <2 hrs	1.43 (1.00 to 2.03; 0.048)		1.08 (0.76 to 1.51; 0.68)	
≥2 hrs	1.37 (0.96 to 1.94; 0.081)		0.89 (0.64 to 1.23; 0.48)	

Legend: Questionnaire imputed sample: n = 8,954 (weighted sample: n = 6,976). Time-use-diary imputed sample: n = 2,520 (weighted sample: n = 5,727). [a] Adjusted for sex, ethnicity, religion, peer alcohol use, parental alcohol use, parental cigarette use, parental e-cigarette use, parenting style, previous cigarette use, previous e-cigarette use, anti-social behaviour, previous alcohol use, urbanicity, age, number of siblings in household, maternal age at participant birth, in-person activities, cognitive ability, mental health, and risk-taking. Values may not add up due to rounding. Abbreviations: CI = Confidence interval; Hr/s = Hour/s; Min/s = Minute/s; n = Number of participants; Ref = Reference category, and RR = Risk ratio.

Table-H4. Participant binge drinking according to time spent on social media, within strata of parental education and according to 'combinations' of time spent on social media and parental education within the questionnaire and time-use-diary complete case samples

	**Questionnaire complete case sample (n = 5,317)**		**Time-use-diary complete case sample (n = 1,826)**	
	**High parental education**	**Low parental education**	**High parental education**	**Low parental education**
**Outcome: Binge drinking**				
**Weighted prevalence % (observed n with outcome/without outcome)**				
No social media use	29.3 (78/188)	35.4 (31/85)	54.2 (296/305)	54.7 (119/111)
1 - <30 mins	49.6 (205/255)	39.7 (79/132)	65.0 (159/104)	51.3 (44/48)
30 mins - <1 hr	59.3 (307/243)	54.5 (123/138)	61.5 (126/83)	44.2 (40/45)
1 - <2 hrs	64.4 (387/213)	55.5 (162/163)	56.7 (82/51)	61.6 (41/26)
≥2 hrs	71.0 (991/480)	65.5 (642/415)	48.5 (45/38)	57.3 (34/29)
**Unadjusted RR (95% CI; *p-value*) for time spent on social media within strata of parental education**				
No social media use	Ref	Ref	Ref	Ref
1 - <30 mins	1.69 (1.32 to 2.18; <0.0001)	1.12 (0.75 to 1.67; 0.57)	1.20 (1.04 to 1.38; 0.011)	0.94 (0.67 to 1.32; 0.71)
30 mins - <1 hr	2.03 (1.60 to 2.57; <0.0001)	1.54 (1.06 to 2.24;.0.024)	1.14 (0.98 to 1.31; 0.087)	0.81 (0.58 to 1.13; 0.21)
1 - <2 hrs	2.20 (1.77 to 2.74; <0.0001)	1.57 (1.07 to 2.29; 0.020)	1.05 (0.78 to 1.41; 0.76)	1.13 (0.84 to 1.51; 0.42)
≥2 hrs	2.43 (1.95 to 3.02; <0.0001)	1.85 (1.30 to 2.64; 0.001)	0.90 (0.67 to 1.20; 0.45)	1.05 (0.73 to 1.49; 0.80)
**Unadjusted RR (95% CI; *p-value*) for time spent on social media and parental education**				
No social media use	0.83 (0.55 to 1.23; 0.35)	Ref	0.99 (0.81 to 1.22; 0.93)	Ref
1 - <30 mins	1.40 (0.98 to 2.01; 0.068)	1.12 (0.75 to 1.67; 0.57)	1.19 (0.97 to 1.45; 0.089)	0.94 (0.67 to 1.32; 0.72)
No social media use	0.83 (0.55 to 1.23; 0.35)	Ref	0.99 (0.81 to 1.22; 0.93)	Ref
30 mins - <1 hr	1.68 (1.17 to 2.40; 0.005)	1.54 (1.06 to 2.24; 0.024)	1.12 (0.91 to 1.39; 0.28)	0.81 (0.58 to 1.13; 0.21)
No social media use	0.83 (0.55 to 1.23; 0.35)	Ref	0.99 (0.81 to 1.22; 0.93)	Ref
1 - <2 hrs	1.82 (1.28 to 2.59; 0.001)	1.57 (1.07 to 2.29; 0.020)	1.04 (0.74 to 1.45; 0.83)	1.13 (0.84 to 1.51; 0.42)
No social media use	0.83 (0.55 to 1.23; 0.35)	Ref	0.99 (0.81 to 1.22; 0.93)	Ref
≥2 hrs	2.01 (1.42 to 2.84; <0.0001)	1.85 (1.30 to 2.64; 0.001)	0.89 (0.66 to 1.19; 0.43)	1.05 (0.73 to 1.49; 0.80)
**Unadjusted measure of multiplicative effect modification and interaction (95% CI; *p-value*)**				
No social media use	Ref		Ref	
1 - <30 mins	1.51 (0.94 to 2.42; 0.086)		1.28 (0.88 to 1.85; 0.19)	
30 mins - <1 hr	1.31 (0.86 to 2.02; 0.21)		1.40 (0.99 to 1.99; 0.055)	
1 - <2 hrs	1.40 (0.92 to 2.14; 0.12)		0.93 (0.61 to 1.42; 0.73)	
≥2 hrs	1.31 (0.87 to 1.97; 0.19)		0.85 (0.54 to 1.35; 0.50)	

**Adjusted[a] RR (95% CI; *p-value*) for time spent on social media within strata of parental education**				
No social media use	Ref	Ref	Ref	Ref
1 - <30 mins	1.60 (1.26 to 2.03; <0.0001)	1.12 (0.82 to 1.53; 0.48)	1.17 (1.02 to 1.35; 0.021)	0.89 (0.68 to 1.17; 0.40)
30 mins - <1 hr	1.96 (1.56 to 2.47; <0.0001)	1.47 (1.06 to 2.02; 0.019)	1.19 (1.04 to 1.36; 0.014)	0.87 (0.63 to 1.20; 0.39)
1 - <2 hrs	2.15 (1.72 to 2.69; <0.0001)	1.40 (1.01 to 1.94; 0.043)	1.05 (0.81 to 1.37; 0.69)	1.12 (0.86 to 1.47; 0.40)
≥2 hrs	2.44 (1.97 to 3.01; <0.0001)	1.78 (1.30 to 2.44; <0.0001)	1.03 (0.77 to 1.37; 0.86)	1.11 (0.84 to 1.48; 0.45)
**Adjusted[a] RR (95% CI; *p-value*) for time spent on social media and parental education**				
No social media use	0.72 (0.52 to 1.00; 0.047)	Ref	0.96 (0.79 to 1.17; 0.71)	Ref
1 - <30 mins	1.15 (0.86 to 1.54; 0.35)	1.12 (0.82 to 1.53; 0.48)	1.13 (0.94 to 1.36; 0.19)	0.89 (0.68 to 1.17; 0.40)
No social media use	0.74 (0.52 to 1.04; 0.084)	Ref	0.97 (0.80 to 1.17; 0.73)	Ref
30 mins - <1 hr	1.45 (1.08 to 1.95; 0.014)	1.47 (1.06 to 2.02; 0.019)	1.15 (0.93 to 1.41; 0.19)	0.87 (0.63 to 1.20; 0.39)
No social media use	0.75 (0.54 to 1.04; 0.084)	Ref	0.98 (0.80 to 1.19; 0.83)	Ref
1 - <2 hrs	1.61 (1.20 to 2.16; 0.002)	1.40 (1.01 to 1.94; 0.043)	1.03 (0.76 to 1.40; 0.84)	1.12 (0.86 to 1.47; 0.40)
No social media use	0.74 (0.52 to 1.06; 0.11)	Ref	0.96 (0.79 to 1.17; 0.67)	Ref
≥2 hrs	1.82 (1.34 to 2.46; <0.0001)	1.78 (1.30 to 2.44; <0.0001)	0.98 (0.74 to 1.30; 0.90)	1.11 (0.84 to 1.48; 0.45)
**Adjusted[a] measure of multiplicative effect modification and interaction (95% CI; *p-value*)**				
No social media use	Ref		Ref	
1 - <30 mins	1.43 (0.96 to 2.11; 0.075)		1.32 (0.97 to 1.78; 0.073)	
30 mins - <1 hr	1.34 (0.93 to 1.93; 0.12)		1.37 (0.97 to 1.94; 0.078)	
1 - <2 hrs	1.53 (1.07 to 2.19; 0.019)		0.94 (0.65 to 1.36; 0.74)	
≥2 hrs	1.37 (0.95 to 1.98; 0.095)		0.92 (0.63 to 1.35; 0.68)	

Legend: Questionnaire complete case sample: n = 5,317 (weighted sample: n = 4,095). Time-use-diary complete case sample: n = 1,826 (weighted sample: n = 3,902). [a] Adjusted for sex, ethnicity, religion, peer alcohol use, parental cigarette use, parental e-cigarette use, parenting style, previous cigarette use, previous e-cigarette use, anti-social behaviour, previous alcohol use, urbanicity, age, number of siblings in household, maternal age at participant birth, in-person activities, cognitive ability, mental health, and risk-taking. Values may not add up due to rounding. Abbreviations: CI = Confidence interval; Hr/s = Hour/s; Min/s = Minute/s; n = Number of participants; Ref = Reference category, and RR = Risk ratio.

REFERENCES

1. Atkin AJ, Dainty JR, Dumuid D, et al. Adolescent time use and mental health: A cross-sectional, compositional analysis in the Millennium Cohort Study. *BMJ Open*. 2021;11(10):e047189. DOI: 10.1136/bmjopen-2020-047189.

2. Fitzsimons E, Villadsen A. Substance use and antisocial behaviour in adolescence. Evidence from the Millennium Cohort Study at age 17. London, UK: Centre for Longitudinal Studies, Institute of Education, University College London; 2021. Available from: https://cls.ucl.ac.uk/wp-content/uploads/2017/02/CLS-briefing-paper-Risky-behaviours-MCS-Age-17-initial-findings.pdf [accessed 26 January, 2023]

3. Twenge JM, Spitzberg BH, Campbell WK. Less in-person social interaction with peers among U.S. adolescents in the 21st century and links to loneliness. *J Soc Pers Relat.* 2019;36(6):1892–913. DOI: 10.1177/0265407519836170

4. Fitzsimons E, Haselden L, Smith K, et al. Millennium Cohort Study: age 17 sweep (MCS7) (second edition). London, UK: Centre for Longitudinal Studies, Institute of Education, University College London; 2020. Available from: https://cls.ucl.ac.uk/wp-content/uploads/2022/05/MCS7-user-guide-Age-17-ed2.pdf [accessed 05 May, 2022]

5. Johnson DR. Using weights in the analysis of survey data. Pennsylvania, USA; 2008. Available from: https://pages.nyu.edu/jackson/design.of.social.research/Readings/Johnson.pdf [accessed 05 August, 2022]

6. Mostafa T, Ploubidis G. Millennium Cohort Study - Sixth survey 2015-2016 technical report on response (Age 14). London, UK: Centre for Longitudinal Studies, Institute of Education, University College London; 2017. Available from: https://doc.ukdataservice.ac.uk/doc/8156/mrdoc/pdf/mcs6_report_on_response.pdf [accessed 04 May, 2022]

7. Social Science Computing Operative. Multiple imputation in Stata. Wisconsin, USA; 2013. Available from: https://www.ssc.wisc.edu/sscc/pubs/stata_mi_intro.html [accessed 04 October, 2023]

8. Knol MJ, VanderWeele TJ, Groenwold RHH, et al. Estimating measures of interaction on an additive scale for preventive exposures. *Eur J Epidemiol*. 2011;26(6):433–8. DOI: 10.1007/s10654-011-9554-9

9. Knol MJ, VanderWeele TJ. Recommendations for presenting analyses of effect modification and interaction. *Int J Epidemiol*. 2012;41(2):514–20. DOI: 10.1093/ije/dyr218

10. Vanderweele TJ. On the distinction between interaction and effect modification. *Epidemiology*. 2009;20(6):863–71. DOI: 10.1097/ede.0b013e3181ba333c
