# Supplementary material for: The relationship between time spent on social media and adolescent alcohol use: a longitudinal analysis of the UK Millennium Cohort Study
Source: Eur J Public Health. 2023 Sep 12;33(6):1043–51. doi: 10.1093/eurpub/ckad163 (PMC10710344; doi:10.1093/eurpub/ckad163)
Supplement: ckad163_Supplementary_Data [file ckad163_supplementary_data.zip › ckad163_Supplementary_Data/ejph-2023-06-om-0290-File006.docx]

# STROBE Statement- Checklist of items that should be included in reports of cohort studies

|  | Item No | Recommendation | Page No |
| --- | --- | --- | --- |
| **Title and abstract** | 1 | (*a*) Indicate the study’s design with a commonly used term in the title or the abstract | P1-2 |
|  |  | (*b*) Provide in the abstract an informative and balanced summary of what was done and what was found | P1-3 |
| Introduction | | | |
| Background/rationale | 2 | Explain the scientific background and rationale for the investigation being reported | P4-5 |
| Objectives | 3 | State specific objectives, including any prespecified hypotheses | P4-5 |
| Methods | | | |
| Study design | 4 | Present key elements of study design early in the paper | P6 |
| Setting | 5 | Describe the setting, locations, and relevant dates, including periods of recruitment, exposure, follow-up, and data collection | P6  Figure-1 |
| Participants | 6 | (*a*) Give the eligibility criteria, and the sources and methods of selection of participants. Describe methods of follow-up | P6  Figure-1 |
|  |  | (*b*) For matched studies, give matching criteria and number of exposed and unexposed | Not applicable |
| Variables | 7 | Clearly define all outcomes, exposures, predictors, potential confounders, and effect modifiers. Give diagnostic criteria, if applicable | P7-9  Figure-2  Appendix-B |
| Data sources/ measurement | 8* | For each variable of interest, give sources of data and details of methods of assessment (measurement). Describe comparability of assessment methods if there is more than one group | P7-9  Appendix-B |
| Bias | 9 | Describe any efforts to address potential sources of bias | P9-11  Figure-2  Appendix-C  Appendix-D  Appendix-E |
| Study size | 10 | Explain how the study size was arrived at | Figure-1  P11 |
| Quantitative variables | 11 | Explain how quantitative variables were handled in the analyses. If applicable, describe which groupings were chosen and why | P7-9  Appendix-B |
| Statistical methods | 12 | (*a*) Describe all statistical methods, including those used to control for confounding | P9-11  Appendix-C  Appendix-D  Appendix-E |
|  |  | (*b*) Describe any methods used to examine subgroups and interactions | P10-11  Appendix-G |
|  |  | (*c*) Explain how missing data were addressed | P9  Appendix-D |
|  |  | (*d*) If applicable, explain how loss to follow-up was addressed | Figure-1  P9  Appendix-C |
|  |  | (*e*) Describe any sensitivity analyses | P10-11  Appendix-E |
| Results | | |  |
| Participants | 13* | (a) Report numbers of individuals at each stage of study—eg numbers potentially eligible, examined for eligibility, confirmed eligible, included in the study, completing follow-up, and analysed | Figure-1  P11 |
|  |  | (b) Give reasons for non-participation at each stage | Figure-1 |
|  |  | (c) Consider use of a flow diagram | Figure-1 |
| Descriptive data | 14* | (a) Give characteristics of study participants (eg demographic, clinical, social) and information on exposures and potential confounders | P11  Appendix-F |
|  |  | (b) Indicate number of participants with missing data for each variable of interest | Appendix-D  Figure-1 |
|  |  | (c) Summarise follow-up time (eg, average and total amount) | Figure-1 |
| Outcome data | 15* | Report numbers of outcome events or summary measures over time | P11-14  Table-1  Appendix-G  Appendix-H |
